# Network toxicology and single-cell analysis reveal key gene-mediated bisphenol a interference with granulosa cell function in polycystic ovary syndrome

**DOI:** 10.3389/fphar.2026.1754568

**Published:** 2026-03-02

**Authors:** Yan Zhang, Yuan Lin, Xiumei Xiong, Xiujuan Chen, Xiaoqing Liu, Hailong Huang

**Affiliations:** 1 Department of Obstetrics and Gynecology, Fujian Maternity and Child Health Hospital, College of Clinical Medicine for Obstetrics and Gynecology and Pediatrics, Fujian Medical University, Fuzhou, China; 2 Fujian Provincial Key Laboratory of Prenatal Diagnosis and Birth Defect, Medical Genetic Diagnosis and Therapy Center of Fujian Maternity and Child Health Hospital, College of Clinical Medicine for Obstetrics and Gynecology and Pediatrics, Fujian Medical University, Fuzhou, China

**Keywords:** diagnostic biomarkers, endocrine disruptors, granulosa cell apoptosis, network toxicology, polycystic ovary syndrome, single-cell sequencing

## Abstract

**Background:**

Bisphenol A (BPA), a typical endocrine-disrupting chemical, is implicated in the pathogenesis of Polycystic Ovary Syndrome (PCOS); however, the underlying molecular mechanisms and pathophysiological processes remain unclear. This study aims to decipher molecular interactions between BPA and PCOS-related genetic networks, and to determine the combinatorial impacts of environmental pollutants on PCOS progression.

**Methods:**

We first identified overlapping genes associated with bisphenol A (BPA) exposure and polycystic ovary syndrome (PCOS) using the Comparative Toxicogenomics Database (CTD). Differentially expressed genes (DEGs) were extracted from three Gene Expression Omnibus (GEO) datasets, while oxidative stress- and apoptosis-related genes were retrieved from the GeneCards database. Subsequently, a series of *in silico* analyses were performed, including protein-protein interaction (PPI) network construction, functional enrichment profiling, Gene Set Enrichment Analysis (GSEA), immune infiltration evaluation, nomogram development, CB-DOCK molecular docking, and single-cell RNA-seq analysis of the mouse ovarian dataset GSE268919 (DHEA-induced PCOS-like model) to provide cell-type-resolved evidence. Finally, *in vitro* validation was conducted using primary granulosa cells from PCOS patients and healthy controls, as well as KGN cells, to assess hub gene expression. Functional evaluations were carried out via CCK-8 assay, flow cytometry, quantitative polymerase chain reaction (qPCR), and Western blotting.

**Results:**

We identified 139 hub genes between BPA exposure and PCOS, with enrichment in hormone metabolism, ovarian steroidogenesis, and reproductive signaling pathways—among which the apoptotic pathway was prominently associated with these hub genes, indicating BPA exerts a profound impact on cell survival in PCOS. Five hub genes (PTAFR, RACGAP1, CYP19A1, FSHR, DMD) were pinpointed, and a nomogram integrating these genes showed robust PCOS predictive accuracy. Single-gene GSEA further linked the hub genes to immune modulation, inflammation, and cell apoptosis—validating their functional relevance to apoptotic processes in PCOS. Immune cell infiltration analysis revealed discrepancies between PCOS and control groups, with hub genes correlating with specific immune subsets (e.g., pro-inflammatory cells) that may exacerbate apoptotic signaling in ovarian tissues. Molecular docking demonstrated strong binding affinity between BPA and the protein products of hub genes, suggesting direct BPA-mediated interference with their roles in regulating cell apoptosis. In the mouse ovarian scRNA-seq dataset (GSE268919), we observed cell-type-specific dysregulation of Cyp19a1 and Dmd (mouse gene symbols), with stress/apoptosis signatures enriched in specific ovarian cell populations, thereby providing supportive cell-type localization for the hub-gene–associated phenotypes. *In vitro* validation confirmed dysregulated expression of hub genes in PCOS primary granulosa cells; BPA treatment dose-dependently regulated hub gene expression, inhibited KGN cell proliferation, and significantly induced granulosa cell apoptosis.

**Conclusion:**

BPA exposure disrupts granulosa cell survival in PCOS by driving apoptosis-related molecular reprogramming through key gene regulation, thereby elucidating mechanistic links between environmental pollutants and PCOS progression and highlighting potential molecular targets for intervention.

## 1 Introduction

Polycystic ovary syndrome (PCOS), a common endocrine disorder affecting 5%–20% of women of reproductive age (Escobar-Morreale, 2018), is marked by hyperandrogenism, menstrual disturbances, and polycystic ovarian morphology (Lie Fong et al., 2021). However, the intrinsic mechanisms and pathophysiological underpinnings have not been fully elucidated. Its multifactorial etiology integrates genetic, environmental, epigenetic, and metabolic influences, with emerging evidence identifying endocrine-disrupting chemicals (EDCs)—particularly bisphenols (BPs)—as critical environmental triggers that interact with genetic predispositions via epigenetic mechanisms to drive PCOS pathogenesis (Rosenfield and Ehrmann, 2016; Glueck and Goldenberg, 2019; Ma et al., 2019). Bisphenol A (BPA), widely used in plastics and epoxy resins, is ubiquitous in consumer products and absorbed via oral, inhalatory, or dermal routes. Though analogs like BPS, BPF, and BPAF exist, BPA remains standard, with all variants showing estrogenic and endocrine-disrupting activity (Correia-Sa et al., 2017; Chen et al., 2016; Siracusa et al., 2018). Mechanistically, BPA mimics estrogen, disrupting hormonal pulsatility to impair steroidogenesis, folliculogenesis, and ovarian structure (Zhou et al., 2016). Chronic exposure links to anovulatory PCOS, insulin resistance, and hyperandrogenism, likely via targeting ovarian cells (Bloom et al., 2016). Although these findings highlight the contribution of BPA exposure to PCOS development and ovarian dysfunction, the full mechanistic landscape remains incompletely defined.

Recent studies highlight BPA-induced mitochondrial dysfunction as a key driver of PCOS progression. BPA triggers excessive mitochondrial reactive oxygen species (ROS) production, damaging cellular components, reducing mitochondrial membrane potential, and inducing granulosa cells (GCs) apoptosis—mechanisms directly linking EDC exposure to ovarian dysfunction (Wang et al., 2021). BPA exposure induced PCOS-like ovarian morphology, including reduced ovarian size, increased cyst formation, and decreased antral follicles. By disrupting redox balance, BPA impairs steroidogenesis, folliculogenesis, and oocyte quality, with oxidative stress (OS) levels in granulosa cells correlating with fertility impairment. OS in the ovary promotes follicular developmental stasis, degeneration, and granulosa cell apoptosis, undermining follicular development (Ga et al., 2023). As granulosa cells are critical for folliculogenesis and oocyte maturation, BPA - induced mitochondrial stress via excessive ROS exacerbates ovarian dysfunction, establishing a mechanistic framework for PCOS pathogenesis. These findings underscore mitochondrial dysfunction as a central pathway in BPA-related PCOS, offering therapeutic targets to mitigate EDC-induced reproductive disorders.

Despite growing evidence of BPA as reproductive health disruptor, comprehensive understanding of their molecular mechanisms in PCOS remains limited, with prior research focusing predominantly on hormonal imbalances rather than genetic/molecular interactions or immune modulation (Zhao et al., 2024). Utilizing advances in bioinformatics and genomic technologies offers a chance to thoroughly investigate these complex interactions (Wang et al., 2024). Therefore, using bioinformatics advances, this study clarifies how BPA exacerbate PCOS via molecular, immunological, and mitochondrial pathways. We identified BPA - PCOS shared genes via CTD, analyzed three GEO datasets to find DEGs (integrating oxidative stress/apoptosis genes), and conducted PPI network, enrichment, GSEA, and immune infiltration analyses. A nomogram predicted PCOS risk; AutoDock Vina verified BPA and hub genes interactions. Single-cell data revealed hub genes expression and cell trajectories. Addressing BPA’ role in disrupting PCOS - related pathways, this study enhances understanding of environmental contributions and informs targeted interventions.

## 2 Materials and methods

### 2.1 Hub genes selection for BPA toxicity-related

Based on a comprehensive literature review using databases such as PubMed and Web of Science, we selected structural model of Bisphenol A (BPA) to gain precise insights into their reproductive toxicity. Prior literature searches indicate that BPA demonstrate strongly associated with hormonal axis dysregulation and exhibit marked nonlinear correlations with PCOS (Xu et al., 2024). The Comparative Toxicogenomics Database (CTD; http://ctdbase.org/) was utilized to identify potential hub genes of BPA and screen for shared genes between BPA and PCOS (CBP) (Supplementary Table S1). The research steps are illustrated in Supplementary Figure S1.

### 2.2 Construction of protein-protein interaction network between BPA and PCOS

CBP genes identified from the CTD database were submitted to the STRING platform (https://string-db.org) to retrieve high - confidence protein interactions (confidence score >0.7). The resultant Protein - Protein Interaction (PPI) network was visualized using Cytoscape software (v3.9.1), enabling graphical analysis of molecular associations.

### 2.3 Functional enrichment analysis

To characterize the biological functions and pathways linking BPA to PCOS pathogenesis, we performed Gene Ontology (GO) and Kyoto Encyclopedia of Genes and Genomes (KEGG) pathway enrichment analyses on the CBP gene set using the ClusterProfiler R package (v.4.10.1). Terms with Bonferroni - adjusted p < 0.05 were considered statistically significant, delineating key molecular mechanisms and signaling cascades. Details are provided in Supplementary Table S2.

### 2.4 Identification of PCOS - associated genes

PCOS - related datasets from granulosa cells were retrieved from GEO. Data normalization and standardization were done using the limma R package. Three datasets (GSE34526, GSE10946, GSE102293) formed the primary analysis dataset, while two others (GSE106724, GSE98595) were consolidated and normalized as validation datasets. The limma package (v.3.58.1) was used for differential expression analysis between control and PCOS groups; genes with |logFC| ≥ 0.5 and p < 0.05 were selected as DEGs for further study. Oxidative stress- and apoptosis-related genes were downloaded from GeneCards. Venn diagrams visualized overlaps among CBP, DEGs, and the two phenotype-specific gene sets. Differential analysis results were shown via a ggplot2-generated volcano plot and pheatmap-created heatmap.

### 2.5 Development and validation of a nomogram

A nomogram for predicting PCOS risk was developed using hub genes with the rms (v.6.7.1) package. Its predictive accuracy was assessed through receiver operating characteristic (ROC) curve analysis, which was generated by the pROC (v.1.18.5) and ggplot2 (v.3.5.2) packages. Calibration curve analysis (rms and nomogramFormula packages, v.1.2.0.0) assessed model accuracy, while decision curve analysis evaluated clinical utility and net benefit across threshold probabilities.

### 2.6 Gene set enrichment analysis (GSEA)

Single-gene GSEA was performed using GSEA software to explore the biological functions of BPA-exposure-related genes in PCOS progression. Samples were divided into high- and low-expression groups based on median gene expression levels. Molecular pathways associated with gene expression profiles and phenotypic groupings were analyzed using the “c2.cp.kegg.v7.4.symbols.gmt” subset from the Molecular Signatures Database (MSigDB), with results visualized via ggplot2.

### 2.7 Immune cell infiltration analysis

Immune cell subtype abundances were quantified using single-sample gene set enrichment analysis (ssGSEA) with predefined gene signatures. Spearman correlation analysis was performed to assess relationships between key gene expression levels and ssGSEA-derived immune cell scores, with results visualized using the ggplot2 R package. Additionally, the CIBERSORT algorithm was applied to deconvolute immune cell compositions from bulk gene expression data, comparing PCOS patients to controls. Statistical significance was set at p < 0.05, and intercellular correlations were visualized using heatmaps generated with ggplot2.

### 2.8 Molecular docking

Molecular docking was utilized to evaluate the binding affinity between BPA and five proteins. Three-dimensional structures of BPA were acquired from PubChem (https://pubchem.ncbi.nlm.nih.gov/), whereas crystal structures of five proteins were downloaded from the RCSB PDB (https://www.rcsb.org/). After removing water molecules and small ligands, the CB-DOCK platform (https://cadd.labshare.cn/cb-dock2/) was employed to predict binding sites and affinity for protein-ligand complexes. A protein-ligand blind docking strategy was implemented, integrating cavity detection, docking simulations, and homologous template fitting. Two-dimensional receptor-ligand interaction maps were generated using Discovery Studio software to improve visualization of docking results. Outcomes were visualized and interpreted with PyMOL to clarify structure-activity relationships at the molecular level.

### 2.9 Single-cell RNA sequencing analysis

The scRNA-seq dataset GSE268919 was obtained from a published mouse ovarian PCOS-like model (DHEA-induced) including PCOS-like and control mice (Luo et al., 2024). After standard quality control and preprocessing in Seurat (v5.3.0), cells were filtered according to the published pipeline (e.g., thresholds for UMI/gene counts, mitochondrial content, and dissociation-related gene signatures), followed by normalization and dimensionality reduction. Cell clusters were annotated based on canonical marker genes to define major ovarian cell types, and hub-gene expression patterns were then examined in a cell-type-resolved manner. Notably, gene symbols in the scRNA-seq section follow mouse nomenclature (Cyp19a1, Dmd). Differential expression analysis was performed using FindAllMarkers (min.pct = 0.25, logfc.threshold = 0.25) to identify cluster-specific markers.

### 2.10 Clinical sample collection

Six PCOS patients (diagnosed per Rotterdam criteria) and six healthy controls (undergoing oocyte retrieval for tubal infertility) were enrolled. Exclusion criteria included other ovarian disorders, non-PCOS hyperandrogenism, metabolic/gynecological diseases, and organ dysfunction. Baseline demographic and clinical characteristics of the clinical validation cohort are summarized in Supplementary Table S3. The two groups were generally comparable, with no statistically significant differences in baseline variables except AMH. The study was approved by the Ethics Committee of Fujian Maternity and Child Health Hospital (No. 2025KY222) with written informed consent from all participants, complying with the Declaration of Helsinki. Follicular fluid was collected during oocyte retrieval, centrifuged at 300 × g for 10 min. The supernatant was mixed with 50% Percoll (Solarbio, Beijing) and re-centrifuged at 400 × g for 20 min. Isolated granulosa cells were stored at −80 °C for subsequent experiments.

### 2.11 Cell culture and treatment

The human ovarian granulosa cell line KGN (Shuochengbio, Shanghai) was cultured in DMEM/F-12 (Gibco) supplemented with 10% fetal bovine serum and 1% penicillin-streptomycin (Invitrogen) at 37 °C in a 5% CO_2_ humidified incubator. Bisphenol A (BPA; CAS Number: 80–05-7; purity ≥99%) was acquired from Sigma-Aldrich (St. Louis, MO, United States), and dimethyl sulfoxide (DMSO) was sourced from Sorabi (Beijing, China). For experimental use, BPA was first dissolved in DMSO to prepare a stock solution, which was then further diluted with cell culture medium to achieve the target working concentrations.

### 2.12 Cell viability assay (CCK-8)

Logarithmic-phase KGN cells were seeded into 96-well plates, then treated with BPA at gradient concentrations (0, 10, 50, 100, 150, 200, 250, 300, or 350 μM) for exposure durations of 0, 24 h, 48 h, 72 h, and 96 h, respectively. Post-treatment, 10 μL of CCK-8 reagent (G-Clone, Beijing) was added to each well, followed by a 4-h incubation. The absorbance at 450 nm was measured using a microplate reader, and this reading was used to assess cell viability. We acknowledge that the micromolar concentrations used *in vitro* exceed typical biomonitoring-derived internal exposure levels. In the present study, this concentration gradient was applied as a toxicological/mechanistic *in vitro* dosing range to establish a dose-time response and estimate an IC50 within a short experimental window, which is a common approach in granulosa cell/KGN models when evaluating BPA-related cellular stress, viability and apoptosis phenotypes (Celar Sturm et al., 2025; Tang et al., 2024; Huang et al., 2024; Li et al., 2024). Importantly, this design is not intended to equate acute micromolar exposure with chronic low-dose endocrine disruption; low-dose and long-term exposure paradigms may elicit distinct biological responses and warrant dedicated experimental models (Liu et al., 2021; Rajkumar et al., 2021).

### 2.13 Apoptosis assessment (flow cytometry)

KGN cells (1 × 10^5^/well) were seeded in 6-well plates, treated with BPA(0, 10, 50, or 100 μM, for 24 h), rinsed with PBS, and digested with 0.25% trypsin (Gibco). The concentrations were selected to cover a lower-to-higher toxicological range based on the CCK-8 dose–response and prior literature. Cells were centrifuged at 1000 rpm for 5 min, resuspended in 500 µL 1× Binding Buffer, and stained with 5 µL Annexin V-APC and 10 µL PI (Elabsciences, Wuhan) for 5 min at room temperature in the dark. Apoptotic cells were quantified using a BD Accuri™ C6 flow cytometer.

### 2.14 Quantitative real-time PCR (qPCR)

Total RNA was extracted from KGN cells and primary granulosa cells using TRIzol (Invitrogen), with purity and concentration determined by the A260/A280 ratio. cDNA was synthesized via a Takara Reverse Transcription Kit. qPCR was performed with SYBR GREEN Master Mix (Takara) on an ABI 7500 system. Relative gene expression was calculated using the 2^-△△Ct^ method, normalized to GAPDH. The primer sequences are shown in Table 1.

**TABLE 1:** mRNA - specific primers of genes.

Gene	Primer	Sequence (5′–3′)
GAPDH	FORWARD	GGA​GCG​AGA​TCC​CTC​CAA​AAT
	REVERSE	GGC​TGT​TGT​CAT​ACT​TCT​CAT​GG
PTAFR	FORWARD	TGC​CCT​GGA​CCC​TTG​CTG​AG
	REVERSE	TGC​GGA​ACT​TCT​TGG​TGA​GGA​AAC
RACGAP1	FORWARD	TCC​ACC​CTC​ACC​AAG​AAC​ACT​CC
	REVERSE	GCT​GGG​AAG​TAA​CAG​GCA​GAT​GTG
CYP19A1	FORWARD	ACA​CAT​CTG​GAC​AGG​TTG​GAG​GAG
	REVERSE	CAG​CAT​GAC​ACG​ACG​CAG​AAG​G
FSHR	FORWARD	CCC​TCC​TTG​TGC​TCA​ATG​TCC​TG
	REVERSE	TGG​CGA​TCC​TGG​TGT​CAC​TAG​AG
DMD	FORWARD	ACA​GAG​GGT​GAT​GGT​GGG​TGA​C
	REVERSE	GGG​CAG​CGG​TAA​TGA​GTT​CTT​CC
BCL-2	FORWARD	AGA​TTT​GGC​AGG​GGC​AGA​AAA​CTC
	REVERSE	TGT​GGA​GAG​AAT​GTT​GGC​GTC​TTG
BAX	FORWARD	TCG​CCC​TTT​TCT​ACT​TTG​CCA
	REVERSE	CGG​AGG​AAG​TCC​AAT​GTC​CAG
Caspase3	FORWARD	CAT​GGA​AGC​GAA​TCA​ATG​GAC​T
	REVERSE	CTG​TAC​CAG​ACC​GAG​ATG​TCA

### 2.15 Western blotting

KGN cells or primary granulosa cells were lysed in RIPA buffer on ice for 10 min, and supernatants were collected after centrifugation. Protein concentration was measured via BCA assay. Equal amounts of protein were separated by 10% SDS-PAGE, transferred to PVDF membranes (Millipore), blocked with 5% non-fat milk for 1 h at room temperature, incubated with primary antibodies overnight at 4 °C, and then with secondary antibodies for 1 h at room temperature. Bands were visualized using ECL reagent.

### 2.16 Statistical methods

Statistical analyses were performed using R (v4.3.3), GraphPad Prism (v9.0), and IBM SPSS Statistics (v26.0). Normality and homogeneity of variance were assessed using Shapiro–Wilk and Levene’s tests, respectively. For two-group comparisons, an unpaired two-tailed Student’s t-test was applied to normally distributed data; otherwise, the Mann–Whitney U test was used. For comparisons involving ≥3 groups, one-way ANOVA followed by Dunnett’s multiple-comparisons test versus the control group was used; when distributional assumptions were not met, the Kruskal–Wallis test with Dunn’s *post hoc* correction was applied. Time-course proliferation data were analyzed by two-way ANOVA (time × treatment) with Dunnett’s multiple-comparisons test. Dose–response curves and IC50 values were estimated by nonlinear regression using a four-parameter logistic model. Correlations were evaluated using Spearman’s rank correlation. For bulk transcriptome-derived ssGSEA scores and hub-gene expression comparisons, two-sided Wilcoxon rank-sum tests were used, with Benjamini–Hochberg correction applied where multiple immune cell types were tested simultaneously. ROC AUCs and 95% confidence intervals were computed using the DeLong method. All tests were two-sided, and p < 0.05 was considered statistically significant.

## 3 Results

### 3.1 PPI network construction and enrichment analysis

**FIGURE 1:**
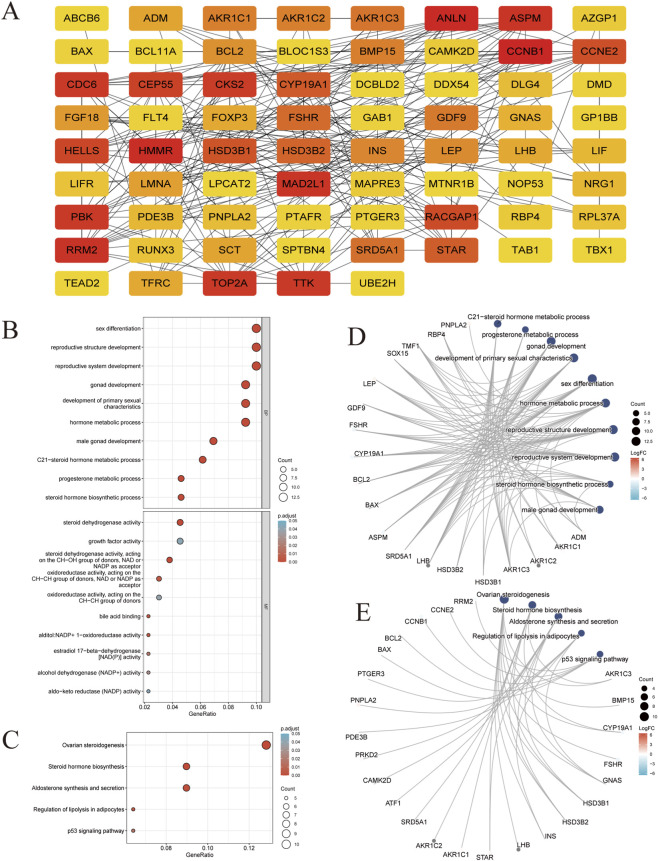
Identification and PPI analysis of hub genes (n = 139 hub genes). **(A)** PPI network of Bisphenols and PCOS hub genes. Each node represents a gene, with the color intensity indicating the degree of connectivity; red nodes have higher connectivity, highlighting them as potential key players in the interaction network. **(B–E)** Enrichment analysis of hub genes in PCOS and Bisphenols. Bubble Chart and Interaction Network Graph illustrated the top 10 enriched entries for each category [GO **(B,D)**, KEGG **(C,E)**] on the 139 potential hub genes. The size of the circles corresponds to the number of genes involved in each process, and the color gradient of the circles represents adjusted p-values, with darker shades indicating higher statistical significance. Note: Adjusted p-values for GO/KEGG enrichment were corrected for multiple testing using the Benjamini–Hochberg method.

We screened 139 shared genetic markers between BPA and PCOS (Figure 1A). PPI network visualization of molecular interactions among these genes revealed an intricate regulatory network reflecting key biological pathways in BPA - induced PCOS pathogenesis. Nodes were color-coded by connectivity degree, with hub genes exhibiting high centrality, suggesting their pivotal roles in network architecture. Functional enrichment analysis (Figures 1B–E) uncovered notable enrichment in hormonal and reproductive pathways. Significantly enriched biological processes included steroid hormone biosynthesis, metabolic processes, secretion, translocation, and regulatory mechanisms. Predominant regulatory pathways such as lipid metabolism, cell growth and death, endocrine metabolic disease, signaling molecules interaction, and endocrine system regulation were also prominent, underscoring their relevance to PCOS pathophysiology. Remarkably, the cell growth and apoptosis signaling pathway emerged as one of the most significantly enriched, highlighting that apoptosis modulation serves as a pivotal mechanism through which BPA drives PCOS pathogenesis. These findings validate that BPA exposure potentially disrupts endocrine homeostasis and reproductive signaling, aligning with the hypothesis that BPA contribute to PCOS progression by interfering with hormonal regulatory networks.

### 3.2 Differential expression and intersection analysis

**FIGURE 2:**
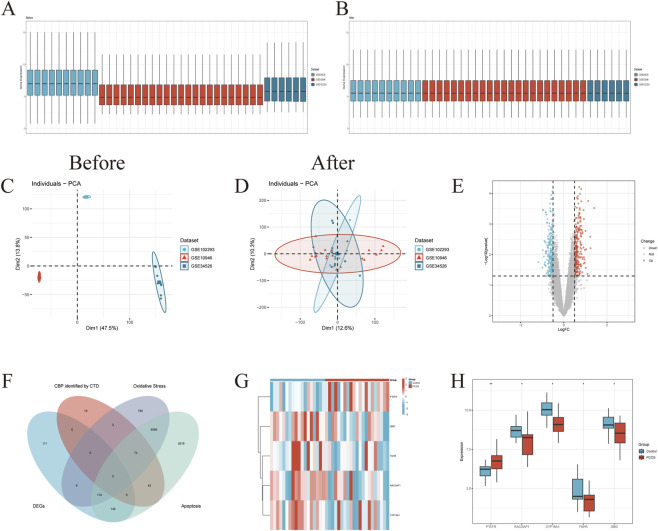
The analysis of DEGs associated with PCOS and BPA. **(A,B)** Gene expression distribution comparison of three datasets (GSE34526, GSE10946, and GSE102293) before **(A)** and after **(B)** integration (n = 33 samples). **(C,D)** Principal Component Analysis (PCA) comparison of three datasets before **(C)** and after **(D)** integration (n = 33 samples). **(E)** A volcano plot illustrating the differential expression analysis of the merged PCOS-related datasets from the GEO database. **(F)** A Venn diagram illustrating the intersection among multiple gene sets: the 448 DEGs from GEO datasets, the 139 hub genes associated with both BPA and PCOS identified by the CTD, as well as genes related to two phenotypes, namely, oxidative stress and apoptosis. **(G)** A heatmap depicting the expression levels of the five hub genes (PTAFR, RACGAP1, CYP19A1, FSHR and DMD) across PCOS and control samples in the combined dataset. **(H)** A boxplot comparing the expression levels of five hub genes between control and PCOS samples. Note: Two-sided Wilcoxon rank-sum test was used to compare gene expression between PCOS and control groups. *p < 0.05, **p < 0.01.

To identify PCOS-related genetic markers, we first integrated three GEO datasets into a unified training set and evaluated batch-effect correction by comparing gene expression distributions and principal component analysis before and after integration (Figures 2A–D). Differential expression analysis of the merged dataset identified 448 differentially expressed genes (DEGs) between PCOS and control samples (Figure 2E). Given that pathway analyses highlighted apoptosis as a key process associated with BPA exposure, we focused on apoptosis-related mechanisms for subsequent analyses. We then intersected the 448 DEGs with 139 BPA-PCOS hub genes retrieved from the CTD and with two phenotype-related gene sets (oxidative stress and apoptosis), yielding five overlapping hub genes: PTAFR, RACGAP1, CYP19A1, FSHR, and DMD (Figure 2F). These genes may represent key molecular links between BPA-associated perturbations and PCOS pathophysiology. A heatmap further illustrated the expression patterns of these five hub genes in PCOS and control samples within the combined dataset (Figure 2G). Boxplot analysis revealed significant between-group differences in hub gene expression (Figure 2H): PTAFR was significantly upregulated in PCOS samples, whereas RACGAP1, CYP19A1, FSHR, and DMD were downregulated. These expression patterns were further supported in an independent validation dataset (Supplementary Figure S2D). Collectively, these findings identify five hub genes as candidate mediators linking BPA-associated molecular perturbations to PCOS-related granulosa cell dysfunction.

### 3.3 Construction and evaluation of a PCOS risk nomogram

**FIGURE 3:**
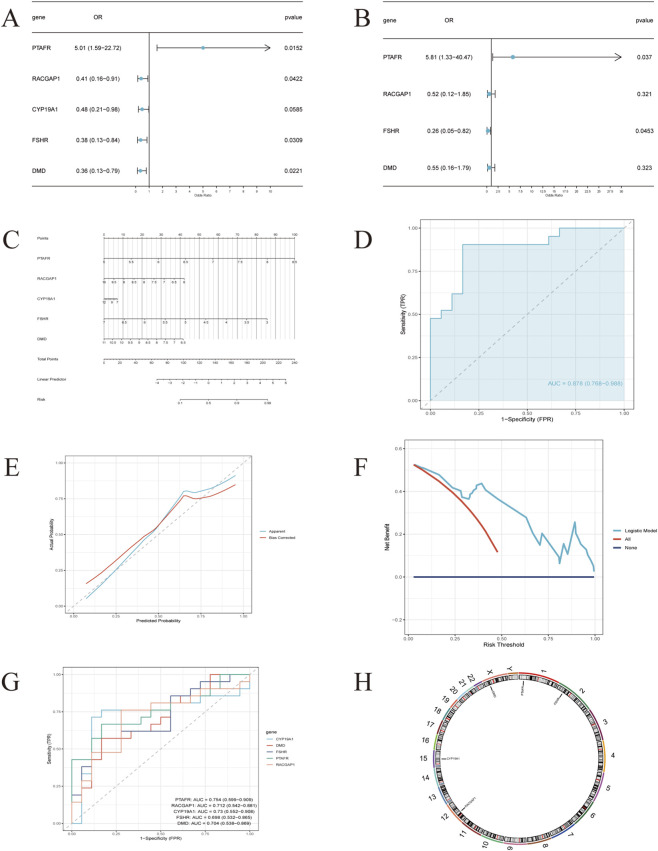
Construction and validation of a nomogram for predicting PCOS risk associated with BPA exposure (n = 139 hub genes). **(A)** Univariate Cox regression analysis of five hub genes (PTAFR, RACGAP1, CYP19A1, FSHR and DMD). **(B)** Multivariate Cox regression analysis of five hub genes. **(C)** A nomogram constructed using five hub genes relevant to BPA exposure. **(D)** A ROC curve evaluating the discriminative power of the nomogram. **(E)** A calibration curve depicting the agreement between the predicted probabilities and actual outcomes of PCOS risk. **(F)** A Decision Curve Analysis (DCA) assessing the clinical utility of the nomogram. **(G)** ROC curves of the five hub genes in the training set. **(H)** Chromosomal localization of the five hub genes. Note: Cox regression p-values were derived from Wald tests; ROC AUCs were calculated with 95% CIs using the DeLong method.

Cox regression analyses explored associations between five hub genes (PTAFR, RACGAP1, CYP19A1, FSHR, DMD) and PCOS risk. Results (Figure 3A) showed PTAFR linked to increased risk, while RACGAP1, FSHR, and DMD were associated with reduced risk. Multivariate Cox regression (Figure 3B) confirmed PTAFR and FSHR as independent predictors in risk stratification. A nomogram integrating the five hub genes was developed to predict PCOS risk in BPA-exposed individuals (Figure 3C). ROC curve analysis (Figure 3D) demonstrated strong discriminative performance with an AUC of 0.878 in the primary set and 0.802 in the validation set (Supplementary Figure S2C), confirming its ability to differentiate cases. The calibration curve (Figure 3E) showed good alignment between predicted and observed outcomes, and decision curve analysis (Figure 3F) validated clinical utility via consistent net benefit across threshold probabilities. These findings support the nomogram as a potential screening tool for BPA-mediated PCOS risk, aiding early intervention. ROC curves for the training set (Figure 3G) assessed the five hub genes’ risk-discriminating ability, and a chromosomal localization map (Figure 3H) illustrated their genomic positions, contextualizing their roles in PCOS pathogenesis.

### 3.4 Single-gene enrichment analysis

To elucidate the potential pathobiological roles of five hub genes in PCOS development, GSEA was conducted (Supplementary Figure S3). PTAFR, enriched in immune cell activation, cytokine signaling, and inflammatory pathways (processes tightly intertwined with apoptosis), may mediate follicular inflammation and hormonal disruption in PCOS by promoting granulosa cell apoptosis via immune dysregulation-driven inflammatory cascades (Supplementary Figure S3A). RACGAP1’s enrichment in cell cycle/proliferation pathways highlights its critical role in regulating cell cycle progression, whose dysregulation disrupts granulosa cell proliferation-apoptosis balance (Supplementary Figure S3B). CYP19A1, a core regulator of steroid hormone biosynthesis and ovarian function linked to ovarian endocrine balance pathways, may indirectly mediate granulosa cell apoptosis via endocrine disruption (Supplementary Figure S3C). FSHR, essential for follicle development and gonadotropin signaling, enriched in ovarian function pathways, may contribute to PCOS anovulation and hormonal dysfunction via impaired FSH-FSHR interactions that disrupt folliculogenesis and trigger granulosa cell apoptosis (Supplementary Figure S3D). DMD—enriched in cancer-related pathways (cell cycle dysregulation, aberrant growth)—may perturb PCOS ovarian cell dynamics by dysregulating granulosa cell proliferation/survival and shifting cell fate toward apoptosis (Supplementary Figure S3E). Collectively, five hub genes converge on granulosa cell apoptosis as a central PCOS pathogenic node, mediating apoptosis via interconnected immune regulation, cell cycle control, and ovarian endocrine signaling (Supplementary Figure S3F) and providing a basis for functional studies on their roles in BPA-induced granulosa cell apoptosis in PCOS.

### 3.5 Immune cell infiltration analysis

**FIGURE 4:**
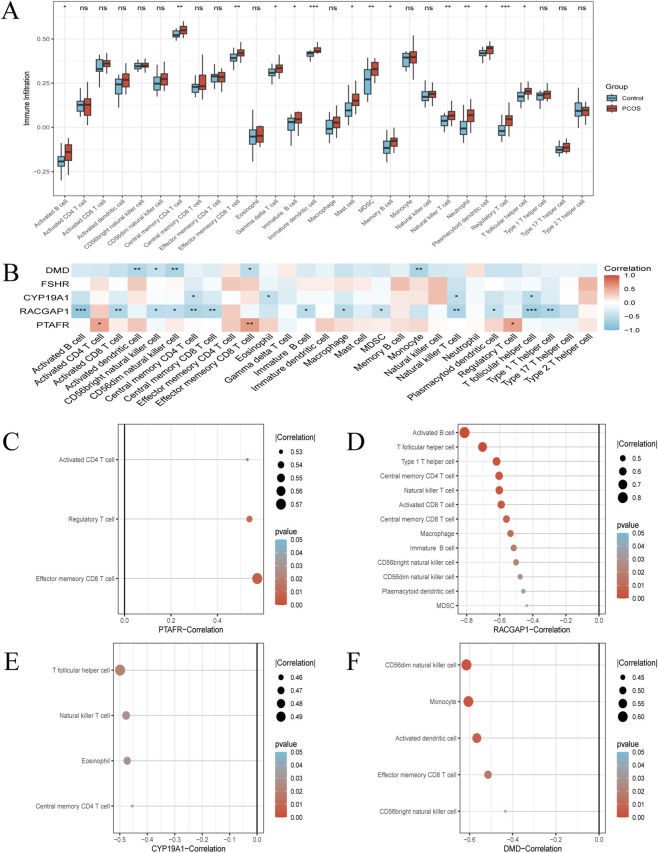
Immune cell infiltration analysis and gene-correlation mapping in PCOS via ssGSEA (n = 5 hub genes). **(A)** Box plots depicting the ssGSEA scores for different immune cell types in control (blue) and PCOS (red) groups (*p < 0.05, **p < 0.01). **(B)** Heatmap representing the correlation coefficients between expression levels of five hub genes (PTAFR, RACGAP1, CYP19A1, FSHR and DMD) and immune cell infiltration levels. The color of the squares indicate the magnitude and direction of the correlation, respectively. **(C–F)** Lollipop plots illustrating correlations between four hub genes [PTAFR **(C)**, RACGAP1 **(D)**, CYP19A1 **(E)**, DMD **(F)**] and immune cell types. Note: Immune cell infiltration was inferred by ssGSEA and CIBERSORT based on bulk transcriptomic data. These results reflect computationally predicted associations and were not functionally validated *in vitro*. **(A)** Two-sided Wilcoxon rank-sum test was used to compare ssGSEA scores between PCOS and control groups. **(B–F)** Correlations were assessed using Spearman’s rank correlation.

Immune cell infiltration patterns and their associations with five hub genes were analyzed to explore immune-related alterations in PCOS (Figure 4). A boxplot (Figure 4A) showed distinct immune profiles between PCOS and control cohorts, with increased estimated infiltration of activated effector memory CD8 T cells, central memory CD4 T cells, immature dendritic cells, B cells, natural killer T cells, neutrophils, and regulatory T cells in PCOS, indicating altered immune signatures in PCOS. Correlation analysis (Figure 4B) revealed significant associations between hub gene expression and predicted immune cell infiltration. Specifically, PTAFR expression was positively correlated with effector memory CD8 T cells, activated CD4 T cells, and regulatory T cells, suggesting a potential link with T-cell activation and immune regulation. RACGAP1 expression showed negative associations with central memory activated B cells and central memory CD4/CD8 T cells, indicating a possible relationship with B-cell activation and T-cell memory status. CYP19A1 expression was negatively correlated with eosinophils and natural killer T cells, suggesting an association with inflammatory and cytotoxic immune components. DMD expression was negatively correlated with activated dendritic cells, implying a potential relationship with antigen-presenting cell activity. In addition, FSHR expression was positively associated with T follicular helper cells, which are involved in follicular immune regulation (Supplementary Figure S4C3). Targeted correlation plots (Figures 4C–F) further illustrated key gene–immune cell associations, highlighting coordinated patterns between hub genes and immune cell signatures in PCOS. Because immune cell infiltration was inferred computationally from bulk transcriptomic data, these results represent association-based, hypothesis-generating evidence rather than experimentally validated immune-mediated mechanisms.

### 3.6 Molecular docking analysis

**FIGURE 5:**
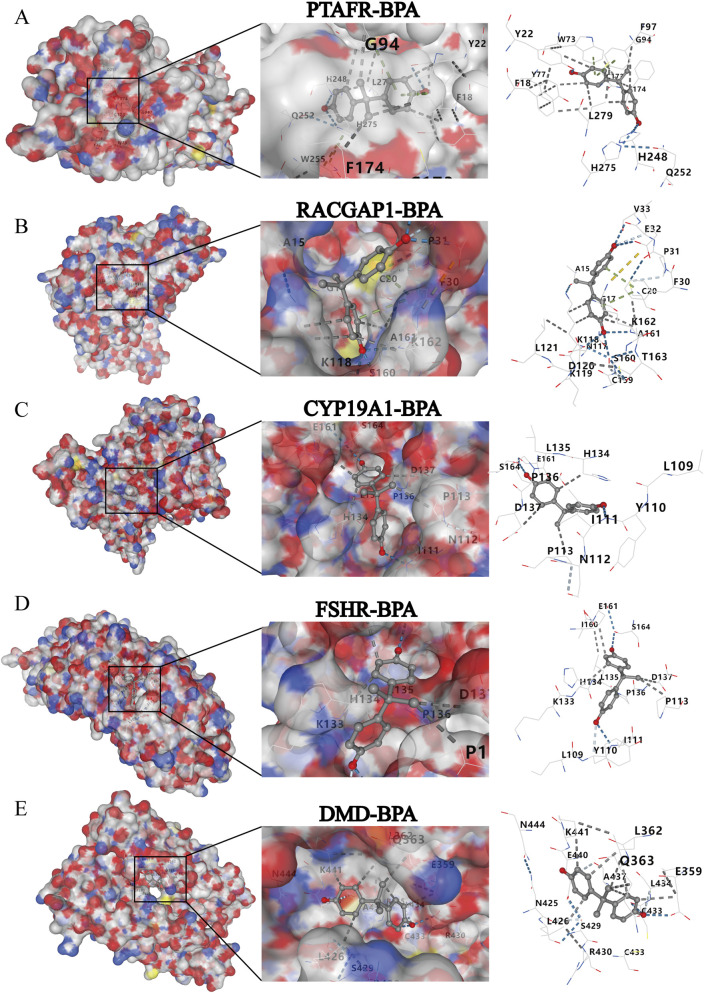
Visualization of molecular docking interactions between five hub genes (PTAFR, RACGAP1, CYP19A1, FSHR, DMD) with BPA. For each hub gene - BPA pair: left panel shows the global protein structure, middle panel zooms in on the local binding site, and right panel details the atomic - level interactions. **(A)** PTAFR - BPA binding mode. **(B)** RACGAP1 - BPA interaction. **(C)** CYP19A1 - BPA docking complex. **(D)** FSHR - BPA binding visualization. **(E)** DMD - BPA docking interaction.

To characterize the crosstalk between five hub genes (PTAFR, RACGAP1, CYP19A1, FSHR, DMD) and BPA compounds, molecular docking analysis was performed. All five proteins demonstrated robust binding to BPA, with docking scores below −6 kcal mol^-1^ (Table 2). Notably, PTAFR exhibited the highest binding affinity to BPA, followed by FSHR and CYP19A1. These findings indicate that BPA may exert the strongest regulatory impact on these hub genes, thereby substantially influencing the pathobiological disruptions characteristic of PCOS. The interaction interfaces between BPA and the five hub proteins were visualized using PyMOL (Figure 5), yielding structural insights at the molecular level into BPA-driven regulation of PCOS-associated gene functions. The results emphasize the potential for BPA exposure to directly influence the activity of five hub genes through specific binding interactions, shedding light on their roles in PCOS pathophysiology.

**TABLE 2:** The result of molecular docking with BPA.

Target	Binding energy (kcal·mol^-1^)
PTAFR	−8.8
RACGAP1	−7.8
CYP19A1	−7.9
FSHR	−8.0
DMD	−7.3

Binding energy is reported in kcal/mol; lower values indicate stronger predicted binding.

### 3.7 Mouse ovarian single-cell transcriptomics analysis

**FIGURE 6:**
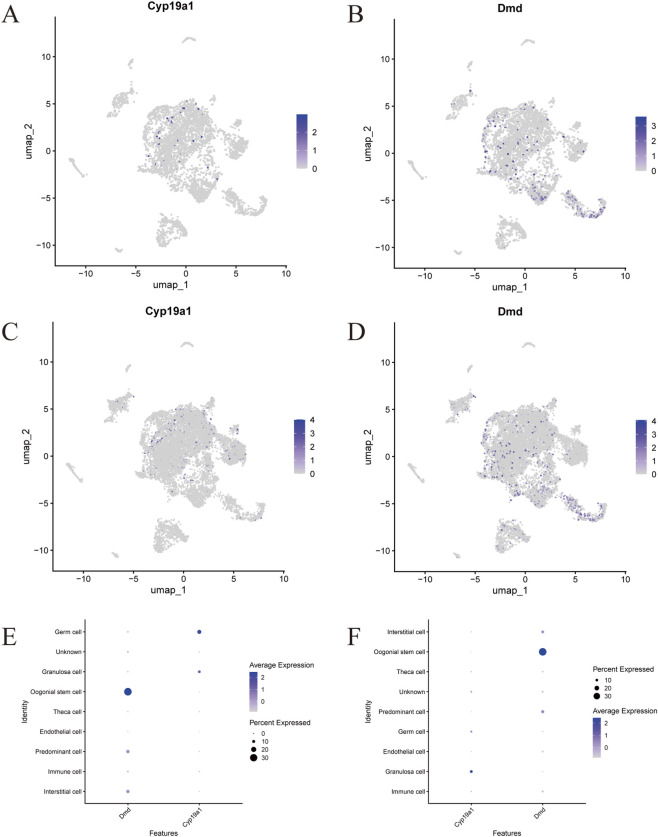
Mouse ovarian scRNA-seq (GSE268919) reveals cell-type-resolved expression of Cyp19a1 and Dmd. **(A,B)** Feature plots showing Cyp19a1 **(A)** and Dmd **(B)** expression in the PCOS-like mouse group. **(C,D)** Feature plots showing Cyp19a1 **(C)** and Dmd **(D)** expression in the control group. **(E,F)** Dot plots summarizing Cyp19a1 and Dmd expression across annotated ovarian cell types in the PCOS-like group **(E)** and controls **(F)**. Note: gene symbols are reported in mouse format (e.g., Cyp19a1, Dmd).

To systematically characterize the ovarian cellular landscape in a mouse PCOS-like model and to localize hub genes dysregulation at single-cell resolution, we analyzed the scRNA-seq dataset GSE268919 (mouse; DHEA-induced PCOS-like model; detailed quality control in Supplementary Figures S5A–D). UMAP clustering identified 22 cell clusters annotated into 9 major types (Supplementary Figures S5E, F), with heterogeneous cell composition between PCOS and control groups (Supplementary Figure S5G, H). Feature plots (Figures 6A–D) revealed cell-type-specific expression: Cyp19a1 was selectively enriched in granulosa and theca cells, while Dmd was specific to germ cells (with reduced expression in PCOS). Dot plots (Figures 6E,F) further quantified this dysregulation: Cyp19a1 expression was significantly elevated in PCOS granulosa cells, whereas Dmd was downregulated in PCOS germ cells. Phenotype analysis underscored the critical connection between these hub genes and granulosa cell apoptosis. Supplementary Figures S7A, B showed distinct clustering of high-stress/apoptosis cells in PCOS, and violin plots (Supplementary Figure S7C, D) confirmed differential apoptotic signature levels between groups. Notably, the high oxidative stress/apoptosis cell populations strongly overlapped with Cyp19a1-expressing granulosa cells—a finding that directly links Cyp19a1 dysregulation to redox imbalance-driven granulosa cell apoptosis. Additionally, elevated AUcell scores for stress/apoptosis phenotypes in PCOS immune and interstitial cells coincided with disrupted Cyp19a1 expression (Supplementary Figure S7A–F), suggesting a synergistic effect of hub gene dysregulation and microenvironmental stress on promoting granulosa cell apoptosis. Collectively, the scRNA-seq analysis demonstrates that hub genes (Cyp19a1 and Dmd) exert cell-type-specific regulatory effects in PCOS, with Cyp19a1 dysregulation in granulosa cells serving as a key molecular link to oxidative stress and apoptotic pathways—a core pathogenic mechanism driving granulosa cell dysfunction and PCOS progression. Dmd downregulation in germ cells, while indirectly related, further supports the broader role of hub gene networks in mediating cellular apoptosis and reproductive impairment in PCOS. Because these scRNA-seq data are derived from a mouse model, we interpret the findings as cell-type-resolved, hypothesis-generating evidence; translational relevance is primarily supported by independent human bulk transcriptomic datasets and validation in human primary granulosa cells.

### 3.8 Experimental verification of hub genes

**FIGURE 7:**
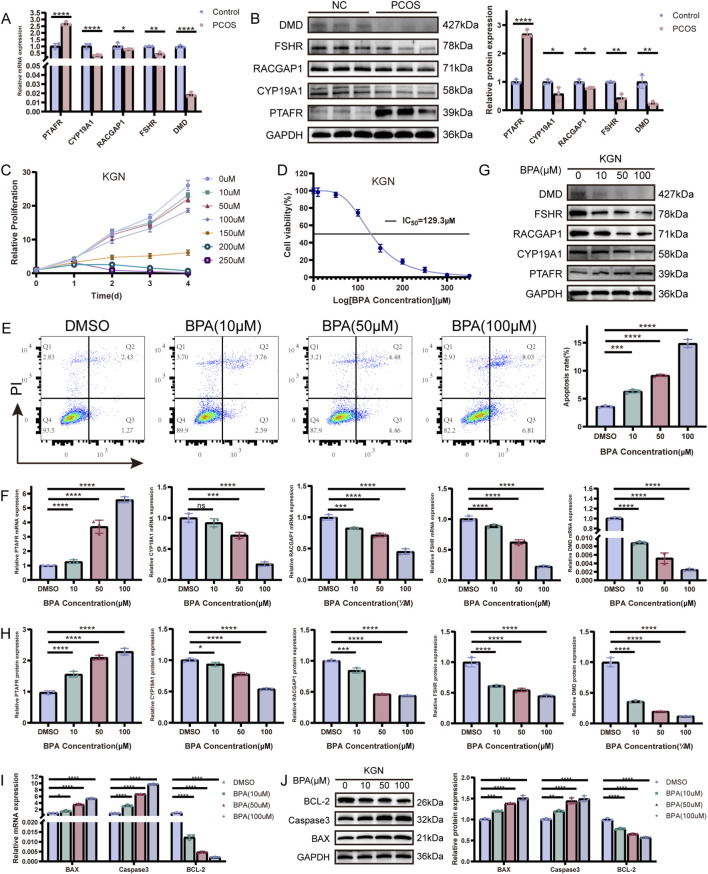
Effects of BPA on molecular expression, proliferation, and apoptosis in ovarian granulosa cells **(A,B)** mRNA **(A)** and protein **(B)** expression of hub genes (PTAFR, RACGAP1, CYP19A1, FSHR, DMD) in primary ovarian granulosa cells from PCOS patients and healthy controls (n = 6 per group; total n = 12). **(C,D)** Proliferation and viability of KGN cells treated with BPA (0, 10, 50, 100, 150, 200, 250, 300, 350 μM): **(C)** Time-dependent relative proliferation; **(D)** CCK-8-derived viability curve with calculated half-maximal inhibitory concentration (IC50 = 129.3 μM). **(E)** Apoptosis rate of BPA-treated KGN cells (0, 10, 50, 100 μM) analyzed by Annexin V-APC/PI double staining via flow cytometry (insets show representative flow plots). **(F–H)** mRNA and protein expression of hub genes in BPA-treated KGN cells (0, 10, 50, 100 μM): **(F)** mRNA expression; **(G)** Protein expression of hub genes; **(H)** Relative protein expression. **(I,J)** mRNA and protein expression of apoptotic-related markers (BAX, Caspase3 and BCL-2) in BPA-treated KGN cells (0, 10, 50, 100 μM). Clinical sample assays were performed in triplicate, and all cell-based experiments were independently repeated three times. Note: All data are presented as mean ± SEM. **(A,B)** Two-sided Mann–Whitney U test (PCOS vs. control; n = 6 per group). **(C)** Two-way ANOVA (time × dose) with Dunnett’s multiple-comparisons test versus 0 μM at each time point. **(D)** IC50 was estimated by nonlinear regression using a four-parameter logistic model. **(E)** One-way ANOVA with Dunnett’s multiple-comparisons test versus 0 μM. **(F,H,J)** One-way ANOVA with Dunnett’s multiple-comparisons test versus 0 μM *p < 0.05, **p < 0.01, ***p < 0.001, ****p < 0.0001.

To validate the biological effects of BPA on ovarian granulosa cells, we first compared hub genes expression in primary granulosa cells from PCOS patients and healthy controls. Results showed significant discrepancies in mRNA and protein levels of PTAFR, RACGAP1, CYP19A1, FSHR, and DMD between the two groups (Figures 7A,B). Functional assays on KGN cells revealed dose- and time-dependent effects of BPA. Time-course proliferation assays demonstrated suppressed cell growth with increasing BPA concentrations (Figure 7C), while CCK-8 viability analysis yielded an IC50 value of 129.3 μM (Figure 7D), indicating reduced viability under toxicological *in vitro* BPA exposure. Flow cytometry showed a significant dose-dependent increase in the apoptosis rate of BPA-treated KGN cells (Figure 7E), with marked elevation at 50 and 100 μM. Molecular analyses of BPA-exposed KGN cells showed dysregulated expression of hub genes: mRNA and protein levels of hub genes (PTAFR, RACGAP1, CYP19A1, FSHR, DMD) were significantly altered in a dose-dependent manner (Figures 7F–H). Additionally, BPA treatment modulated the expression of apoptosis-related markers: BAX and Caspase3 mRNA and protein levels were upregulated, while BCL-2 was downregulated, consistent with the increased apoptotic rate (Figures 7I,J). Collectively, these data demonstrate that toxicological *in vitro* BPA exposure reduces viability, inhibits proliferation, and promotes apoptosis in ovarian granulosa cells, accompanied by dysregulated expression of PCOS-related hub genes and apoptosis signaling pathway components.

## 4 Discussion

Bisphenol A (BPA), a widespread endocrine-disrupting chemical (EDC), perturbs endocrine and metabolic homeostasis (Ahn and Jeung, 2023). Ubiquitous in resins, plastics, and packaging, it exposes humans via dietary intake, skin contact, and inhalation. Epidemiological evidence links BPA exposure to key hallmarks of Polycystic Ovary Syndrome (PCOS), including menstrual irregularities, ovarian dysfunction, and insulin resistance (Patel et al., 2025). Preclinical studies further demonstrate that BPA exposure induces ovarian follicle dysplasia, alters the expression of steroidogenic enzymes, and exacerbates insulin resistance (Javed et al., 2025), while interfering with estrogen signaling, disrupting the hypothalamic-pituitary-ovarian axis, and promoting systemic inflammation. Clinical data also confirm an association between urinary BPA levels and increased PCOS risk (Patel et al., 2024). However, the specific genetic networks mediating BPA-induced granulosa cell apoptosis remain poorly defined, representing a key gap in understanding EDC-related PCOS pathophysiology. This research thus explores the genetic crosstalk between BPA and PCOS, aiming to address this gap and deepen insights into the underlying mechanisms.

Our study identifies a robust association between BPA exposure, dysregulated gene expression in PCOS, and hub genes implicated in both BPA response and PCOS pathogenesis, with five hub genes (PTAFR, RACGAP1, CYP19A1, FSHR, DMD) emerging as core candidates linking BPA-related perturbations to granulosa cell dysfunction. This finding is consistent with prior research on BPA-mediated endocrine disruption (Michenzi et al., 2024), and highlights BPA’s impacts on ovarian function, hormonal balance, and cell survival (Glod et al., 2025; Shi et al., 2021). Clinical validation further indicated that primary granulosa cells from PCOS patients exhibit significantly altered expression of these five hub genes at both mRNA and protein levels compared to healthy controls, supporting their potential involvement in PCOS-related granulosa cell dysfunction. Functional enrichment analysis underscored a strong association between hub genes and apoptosis-related pathways, suggesting that BPA exposure may influence granulosa cell fate decisions. Mechanistically, BPA may interact with nuclear receptors (Gokso Yr et al., 2024) and modulate endocrine homeostasis (Shi et al., 2024), thereby contributing to hub-gene dysregulation and downstream apoptotic signaling (Xu et al., 2022; Zhang et al., 2021). Molecular docking results further supported plausible BPA and hub genes interactions, and *in vitro* assays in KGN cells showed that BPA dose-dependently altered hub-gene expression and apoptosis markers. Taken together, our multi-layer evidence supports a mechanistic link between BPA-associated molecular perturbations and granulosa cell apoptosis in PCOS, while acknowledging that *in vitro* toxicological exposure paradigms do not directly mirror chronic low-dose human exposure.

To provide cell-type-resolved context, we used a PCOS-like mouse ovary scRNA-seq dataset (GSE268919) as an orthogonal resource to localize hub-gene expression across ovarian compartments. Importantly, we did not merge mouse and human expression matrices; the mouse scRNA-seq analysis was used for within-species cell-type mapping and hypothesis generation, whereas differential expression and experimental validation were performed in human cohorts and human granulosa cells. Cross-species ovarian atlases indicate that major ovarian compartments and core steroidogenic programs are broadly conserved across mammals, supporting cautious translational interpretation of hub-gene localization (Wagner et al., 2020; Jones et al., 2024; Morris et al., 2022; Gaylord et al., 2025). Nevertheless, species-specific differences in gene regulation and disease context remain; therefore, we explicitly label the scRNA-seq dataset as mouse and frame these findings as supportive evidence that warrants further validation in human ovarian single-cell datasets when available.

Environmental exposures, as key drivers of PCOS pathogenesis, act as critical modulators that disrupt hormonal homeostasis. BPA exposure suppresses ovarian CYP19A1 expression via ESR1/ESR2 interaction, reducing estrogen biosynthesis and disrupting the hypothalamic-pituitary-ovarian axis to induce hyperandrogenemia and oxidative stress (Mukhopadhyay et al., 2022). This triggers granulosa cell apoptosis via caspase-3 activation, exacerbated by vitamin D deficiency through miR-196b-5p-mediated CYP19A1 suppression (Wan et al., 2021). CYP19A1-driven estrogen reduction enhances androgen-induced ROS, which suppresses CYP19A1 via epigenetic modifications. BPA further amplifies this by inhibiting PI3K/AKT/mTOR-mediated autophagy, exacerbating mitochondrial damage (Guo S. et al., 2025). Therapeutic interventions restore CYP19A1 to mitigate oxidative stress and apoptosis, while MitoQ10 plus vitamin D3 improves follicular maturation in PCOS (Kalimuthu et al., 2025; Kyei et al., 2020). Consistent with these mechanisms, our *in vitro* data demonstrate that BPA dose-dependently downregulates CYP19A1 expression in KGN cells—aligning with impaired estrogen biosynthesis in PCOS. This downregulation was accompanied by elevated apoptotic rates and altered BAX/BCL-2 ratios, supporting the hypothesis that BPA-induced CYP19A1 suppression triggers granulosa cell apoptosis via OS and mitochondrial dysfunction. Collectively, CYP19A1 links BPA exposure to ovarian OS, highlighting its role as a novel therapeutic target for ameliorating environmental and metabolism-related dysregulation with PCOS patients.

BPA interacts with FSHR to form a complex regulatory network that disrupts reproductive health, a key mechanism underlying PCOS pathogenesis. BPA modulates FSHR expression, perturbing hypothalamic-pituitary-ovarian axis homeostasis by reducing aromatase (CYP19A1) activity and enhancing androgen synthesis via CYP17A1 (Mukhopadhyay et al., 2022; Tang et al., 2021). Notably, BPA-induced oxidative stress (OS) directly impairs FSHR function, promoting granulosa cell apoptosis through caspase-3-dependent pathways and transcriptional silencing of Bcl-2 (Mohamed et al., 2025). Elevated OS levels in granulosa cells correlate with reduced FSHR expression—a process further amplified by pro-inflammatory cytokines such as IL-15. IL-15 activates JAK-STAT3 signaling in macrophages, driving M1 polarization and secretion of IL-1β/IL-6, which disrupt FSHR-mediated steroidogenesis via p38 MAPK/JNK phosphorylation (Liu et al., 2022; Chen et al., 2025; Avila et al., 2016). Immune activation exacerbates OS, and heightened OS further impairs FSHR function, amplifying ovarian dysfunction in PCOS. Consistent with our *in vitro* observations, our data demonstrate that BPA treatment decreases FSHR expression in KGN cells, which likely impairs FSH-mediated folliculogenesis and steroidogenesis. Combined with BPA-induced granulosa cell apoptosis, these findings confirm that BPA disrupts FSHR signaling to drive granulosa cell dysfunction—a hallmark of PCOS-related ovarian impairment.

As a G-protein-coupled receptor, the platelet-activating factor receptor (PTAFR) regulates PAF-mediated signaling to control inflammatory and immune responses (Deng et al., 2019). As a widespread endocrine condition, PCOS is defined by chronic low-grade inflammation. An imbalance between Th17 and Treg cells, with a bias toward proinflammatory Th17 cells, contributes to PCOS pathogenesis (Guo Y. et al., 2025), indicated the role of PTAFR takes on additional significance. BPA enhances dendritic cell chemotaxis and IL-23 secretion, potentiating PTAFR-mediated IL-17 production to exacerbate PCOS-related inflammatory milieu (Feng et al., 2025). PCOS is associated with heightened oxidative stress, while BPA exposure is known to induce oxidative stress elevation and induce apoptosis in reproductive tissues, potentially through PTAFR-dependent mechanisms (Jiao et al., 2025). Molecular docking studies confirmed strong binding between BPA and PTAFR, and our *in vitro* data corroborate this interaction: BPA treatment upregulated PTAFR expression in KGN cells and induced apoptosis, effects potentially mediated by enhanced IL-17 production and oxidative stress. Collectively, these findings support a PTAFR-dependent pathway through which BPA exacerbates inflammatory responses and ovarian cell damage in PCOS.

The present results reveal altered immune cell signatures in PCOS and their associations with hub genes expression. Increased estimated infiltration of activated B cells, dendritic cell subsets, and multiple T-cell lineages in PCOS suggests potential interplay between immune dysregulation and endocrine/metabolic disturbance—hallmarks of the disorder. Notably, the immune infiltration patterns and hub gene–immune correlations identified here were inferred from bulk transcriptomic deconvolution and should therefore be interpreted as association-based, hypothesis-generating findings rather than evidence of immune-mediated causality. Within this analytical framework, the observed linkages between hub genes and specific immune cell subsets may indicate that BPA-associated molecular networks intersect with immune-related pathways in PCOS. For example, RACGAP1 showed significant correlations with activated B cells, NKT cells, and Tfh cells, suggesting a potential relationship with inflammatory and adaptive immune signatures that have been reported in PCOS. Likewise, DMD correlated with CD56dim natural killer cells, activated dendritic cells, and monocytes, which may reflect associations with pro-inflammatory immune signatures. These observations are broadly consistent with prior reports describing chronic low-grade inflammation and immune imbalance in PCOS (Shabbir et al., 2023; Wang et al., 2023; Xie et al., 2024). However, because our experimental validation was performed in granulosa cells without an immune microenvironment, future functional studies incorporating immune–granulosa cell co-culture systems, immune-targeted perturbations, or *in vivo* models will be required to determine whether and how immune dysregulation contributes to granulosa cell apoptosis and PCOS-related phenotypes.

Our research used network toxicology, molecular docking, and single-cell analysis, complemented by *in vitro* experiments and clinical sample validation, to investigate BPA-associated molecular perturbations in PCOS. These integrated approaches enabled identification of hub genes and experimental support for apoptosis-related phenotypes, strengthening the interpretability of our findings. However, several limitations should be acknowledged. First, the clinical sample size was relatively small, which may limit the generalizability of hub-gene expression patterns and precludes genetic association analyses (e.g., SNP/GWAS-based gene–environment interaction studies) in the present cohort. Second, although single-cell analysis provided cell-type-resolved context, the scRNA-seq dataset used in this study was derived from a PCOS-like mouse ovarian model; therefore, these findings should be interpreted as supportive, hypothesis-generating evidence, and future studies using human ovarian single-cell/spatial transcriptomic datasets are warranted to strengthen translational relevance. Third, *in vitro* experiments were primarily performed in the KGN cell line and isolated granulosa cells; additional models and broader functional readouts may further strengthen mechanistic depth. In particular, while our data are consistent with mitochondrial stress and apoptosis-related phenotypes, we did not comprehensively characterize mitochondrial function using dedicated assays (e.g., mtDNA copy number, mitochondrial respiration/ATP production, or mitochondrial dynamics markers), nor did we assess hormone-related receptors such as ESR1/ESR2 and LHR that may be relevant to BPA’s estrogen-like activity in granulosa cells. Fourth, while immune infiltration analyses suggested potential immunological associations, the immune component remains bioinformatics-based and was not functionally validated in our granulosa-cell experiments. Finally, the BPA concentrations used for mechanistic interrogation represent toxicological *in vitro* exposure and may not fully recapitulate chronic low-dose human exposure; future longitudinal clinical studies and chronic low-dose experimental designs incorporating endocrine endpoints will be needed to refine exposure thresholds and cell-type-specific responses. Despite these limitations, our work provides a framework linking BPA-associated hub genes to granulosa-cell dysfunction in PCOS and identifies candidate hub genes for further mechanistic and translational validation.

## 5 Conclusion

In summary, this study integrates toxicological network analysis, scRNA-seq, molecular docking, and experimental validation to dissect BPA-induced reprotoxicity in PCOS. Our integrative analyses identify BPA exposure–associated molecular networks and validate five hub genes linked to granulosa-cell apoptosis in PCOS. The findings support a framework in which BPA-associated perturbations relate to ovarian function and cell apoptosis, while immune-related alterations are suggested by bioinformatics analyses. Functional validation of immune mechanisms and chronic low-dose exposure models will be essential in future work. This work uncovers the mechanistic basis of BPA-mediated PCOS pathogenesis, validates hub genes, and lays a foundation for environmental pollutant toxicity assessment and the development of targeted PCOS therapeutics.

## Data Availability

The original contributions presented in the study are included in the article/Supplementary Material, further inquiries can be directed to the corresponding author.

## Supplementary material

SUPPLEMENTARY FIGURE S1 Research flow chart.

SUPPLEMENTARY FIGURE S2 Dataset integration and principal component analysis (PCA) for two datasets(GSE106724, GSE98595) (n = 20 samples). **(A,B)** Gene expression distribution comparison of three datasets before **(A)** and after **(B)** integration. **(C,D)** Principal Component Analysis (PCA) comparison of three datasets before **(C)** and after **(D)** integration. **(E)** ROC curve of the model in the validation set. **(F)** Boxplots illustrate the expression levels of five hub genes (PTAFR, RACGAP1, CYP19A1, FSHR, DMD) in the validation set. Note: **(E)**: AUCs with 95% CIs were calculated using the DeLong method. **(F)**: Two-sided Wilcoxon rank-sum test was used.

SUPPLEMENTARY FIGURE S3 GSEA of hub genes associated with BPA exposure in PCOS. GSEA for PTAFR **(A)**, RACGAP1 **(B)**, CYP19A1 **(C)**, FSHR **(D)** and DMD **(E)** in PCOS. **(F)** Density distribution of pathways related to all genes with BPA exposure in PCOS.

SUPPLEMENTARY FIGURE S4 Immune cell infiltration analysis and gene-correlation mapping in PCOS via CIBERSORT. **(A)** Stacked Bar Plot of immune cell composition in control (blue) and PCOS (red) groups. **(B)** Heatmap representing the correlation coefficients between expression levels of five hub genes (PTAFR, RACGAP1, CYP19A1, FSHR, DMD) and immune cell infiltration levels. The color of the squares indicate the magnitude and direction of the correlation, respectively. **(C1-C4)** Lollipop plots illustrating correlations between four hub genes (PTAFR **(C1)**, RACGAP1 **(C2)**, FSHR **(C3)**, DMD **(C4)** and immune cell types. **(D1-D4)** Scatter plots with linear regression fits for four hub genes (PTAFR **(D1)**, RACGAP1 **(D2)**, FSHR **(D3)**, DMD **(D4)** and their most strongly correlated immune cells. Note: Correlations were assessed using Spearman’s rank correlation; regression lines are shown for visualization.

SUPPLEMENTARY FIGURE S5 Quality control and cell-type annotation of the mouse ovarian scRNA-seq dataset (GSE268919, n = 6 samples). **(A)** Violin plots of key QC metrics (nCount_RNA, nFeature_RNA, log10GenesPerUMI, mitoRatio) in control (blue) vs. PCOS (red) groups. **(B)** PCA-based cell cycle phase distribution (G1: red; G2M: green; S: blue) on PC1-PC2 axes. **(C)** Gene expression variability: residual variance vs. geometric mean of expression, highlighting stable (black) vs. variable (red) genes. **(D)** Correlation of nCount_RNA and nFeature_RNA across cells. **(E)** UMAP of cell clustering after SCT normalization. (F) Cell type annotations (e.g., endothelial, granulosa, immune cells) on UMAP. **(G)** Cell-type proportions in control and PCOS-like mouse ovaries. **(H)** Bar plot showing the cell-type composition in each sample.

SUPPLEMENTARY FIGURE S6 Differential expression analysis of genes in the mouse ovarian scRNA-seq dataset (GSE268919, n = 6 samples). **(A-I)** UMAP-based expression distribution of top 1 differentially expressed gene (DEG) per cell type. Color gradients reflect gene expression levels, highlighting cell-type-specific enrichment patterns. **(J)** Dot plot summarizing expression features of top 5 differential genes per cell type, integrating “Percent Expressed” (dot size) and “Average Expression” (color gradient) to visualize gene activity across cell populations. Note: Differential expression was performed in Seurat using the Wilcoxon rank-sum test with Benjamini–Hochberg correction.

SUPPLEMENTARY FIGURE S7 Single-cell profiling of oxidative stress and apoptosis phenotypes in the mouse ovarian scRNA-seq dataset (n = 6 samples). **(A,B)** UMAP embeddings visualizing AUcell scores for oxidative stress **(A)** and apoptosis **(B)** across all cells. Color intensity reflects score magnitude. **(C,D)** Violin plots depicting distribution of oxidative stress **(C)** and apoptosis **(D)** AUcell scores across major cell types (e.g., immune, granulosa, endothelial cells). **(E,F)** UMAP plots partitioning cells into “high” (red) vs. “low” (blue) groups based on oxidative stress **(E)** and apoptosis **(F)** AUcell scores, illustrating phenotypic distribution patterns.
