# Supplementary figures and images for: Network toxicology and single-cell analysis reveal key gene-mediated bisphenol a interference with granulosa cell function in polycystic ovary syndrome
Source: Front Pharmacol. 2026 Mar 2;17:1754568. doi: 10.3389/fphar.2026.1754568 (PMC12989541; doi:10.3389/fphar.2026.1754568)

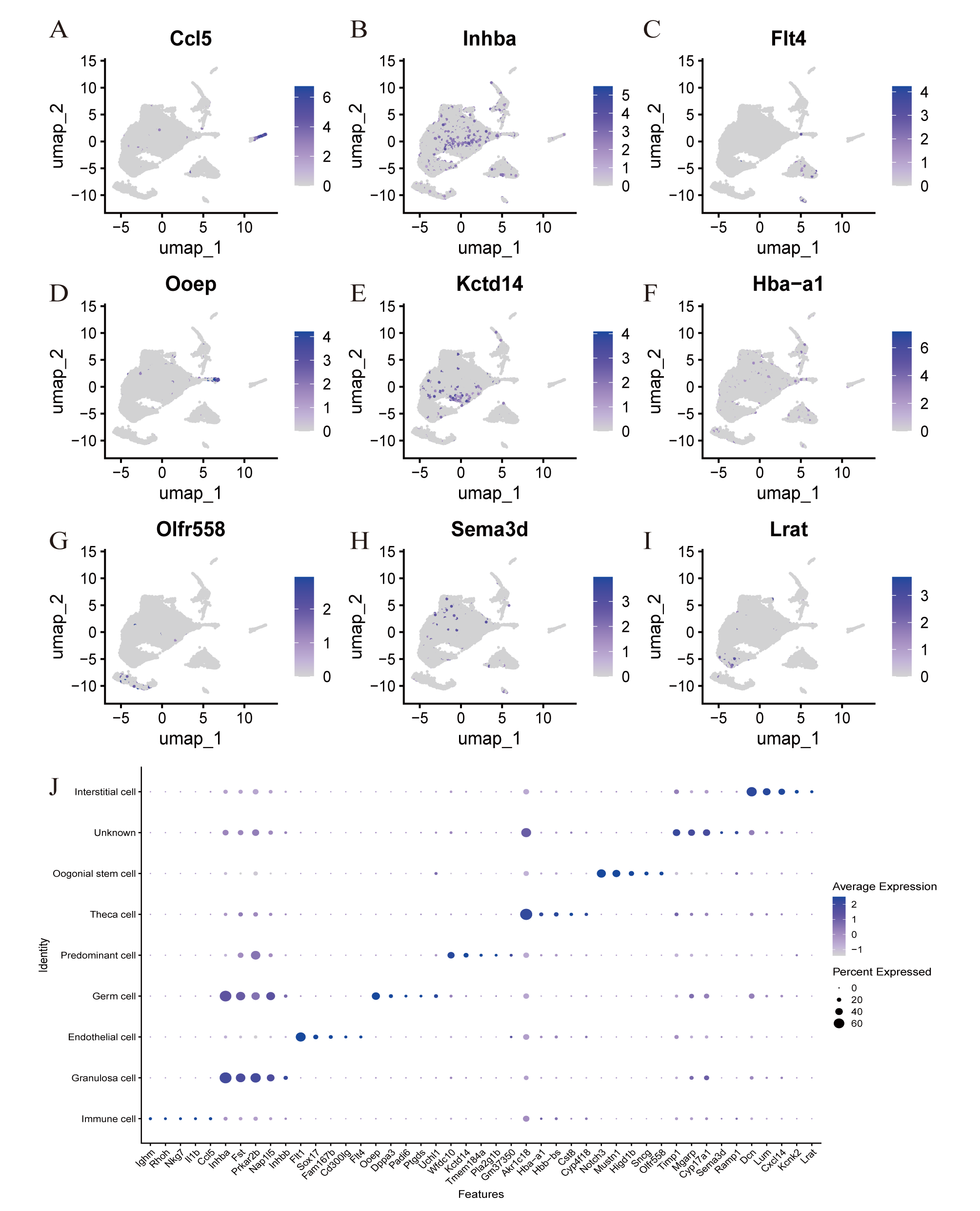

Supplement: Supplementary file 1 [file Image6.tif]

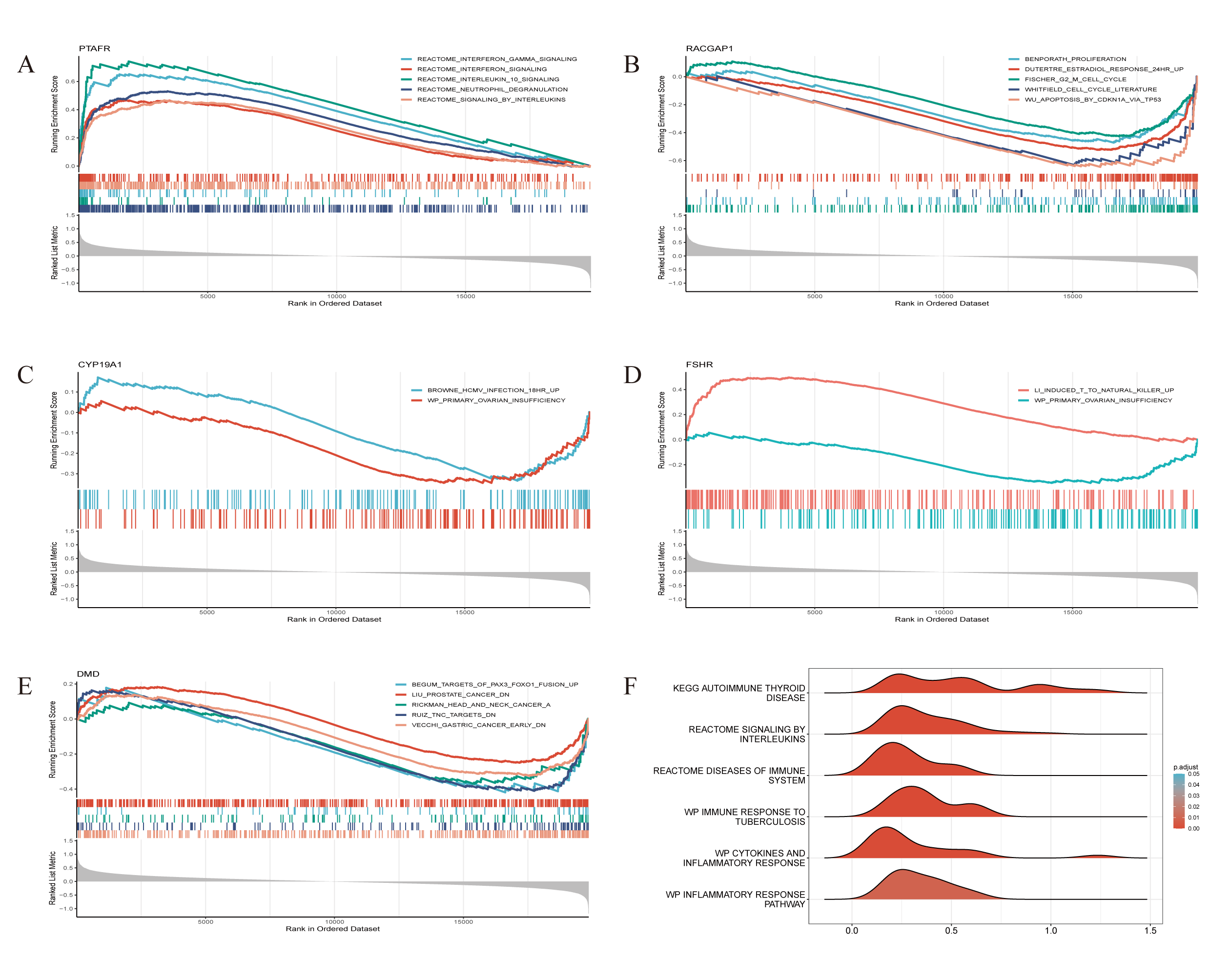

Supplement: Supplementary file 3 [file Image3.tif]

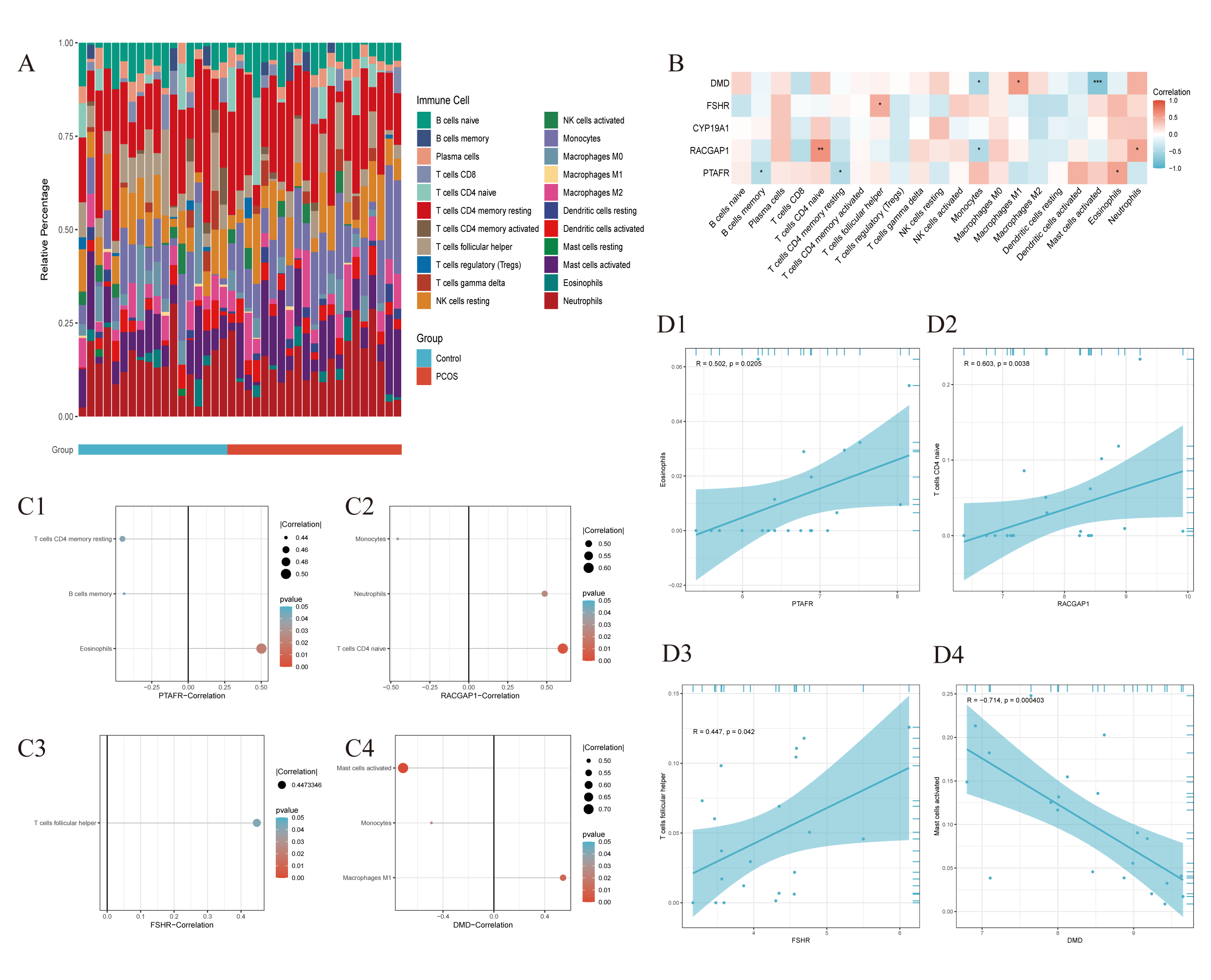

Supplement: Supplementary file 4 [file Image4.tif]

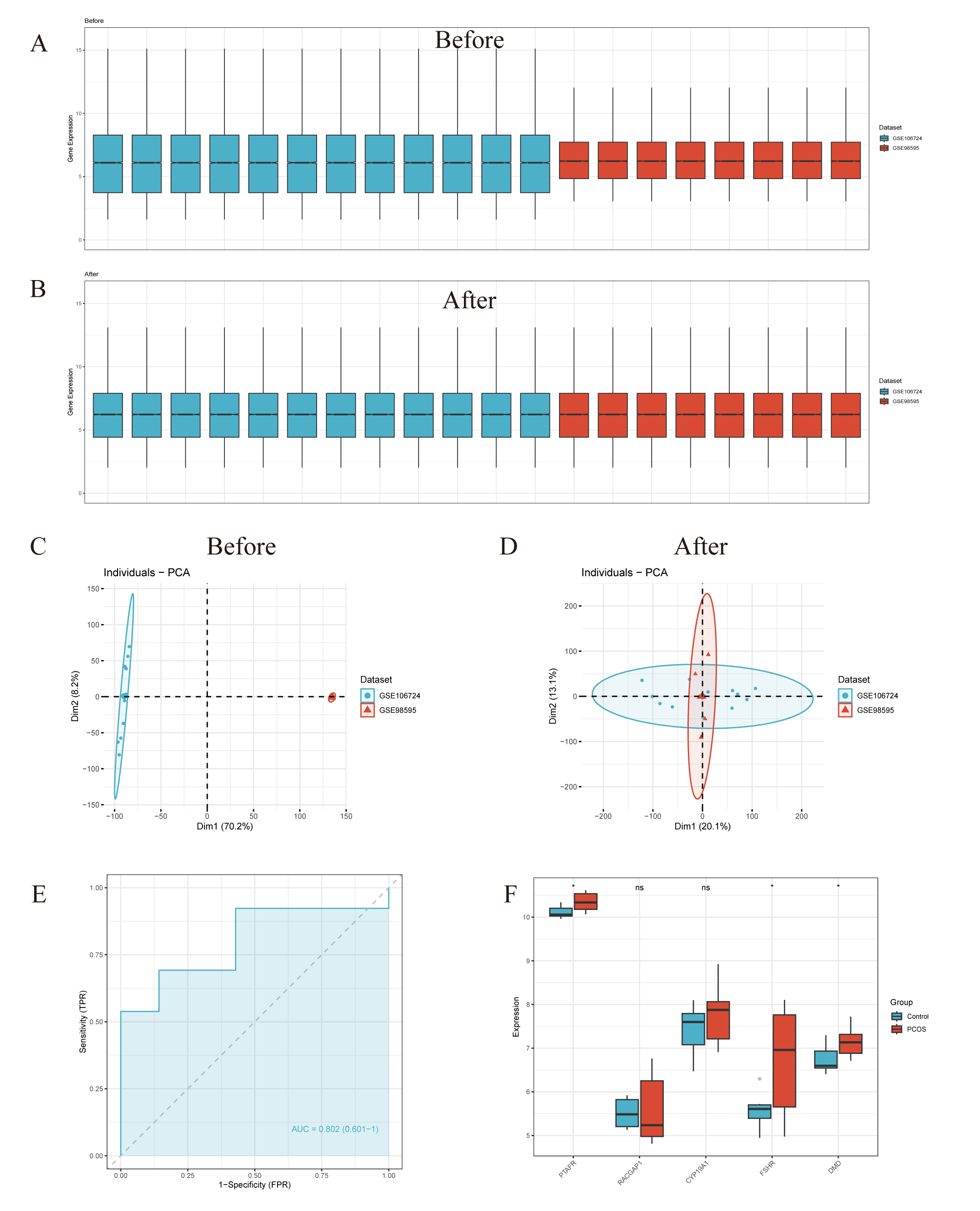

Supplement: Supplementary file 5 [file Image2.tif]

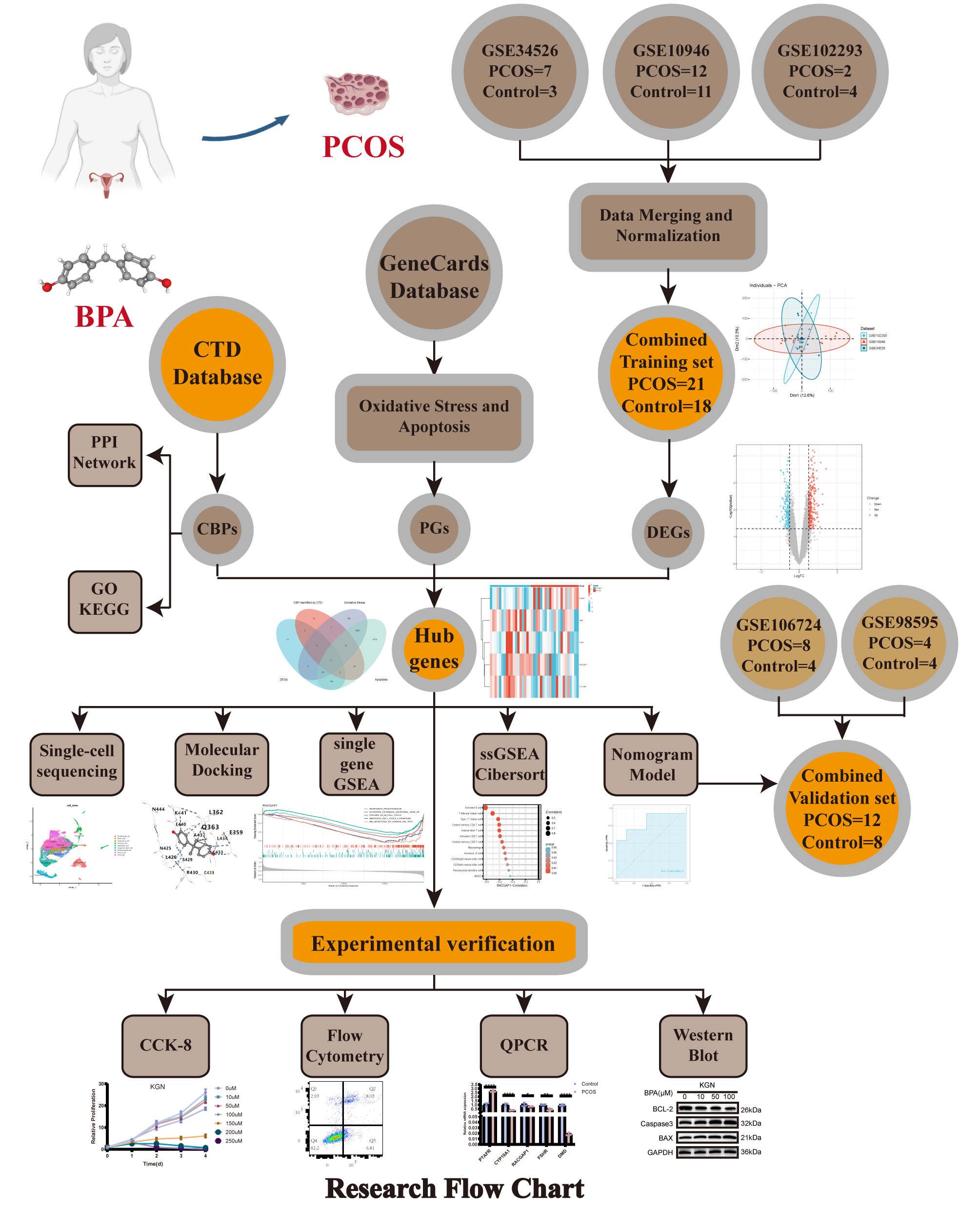

Supplement: Supplementary file 6 [file Image1.tif]

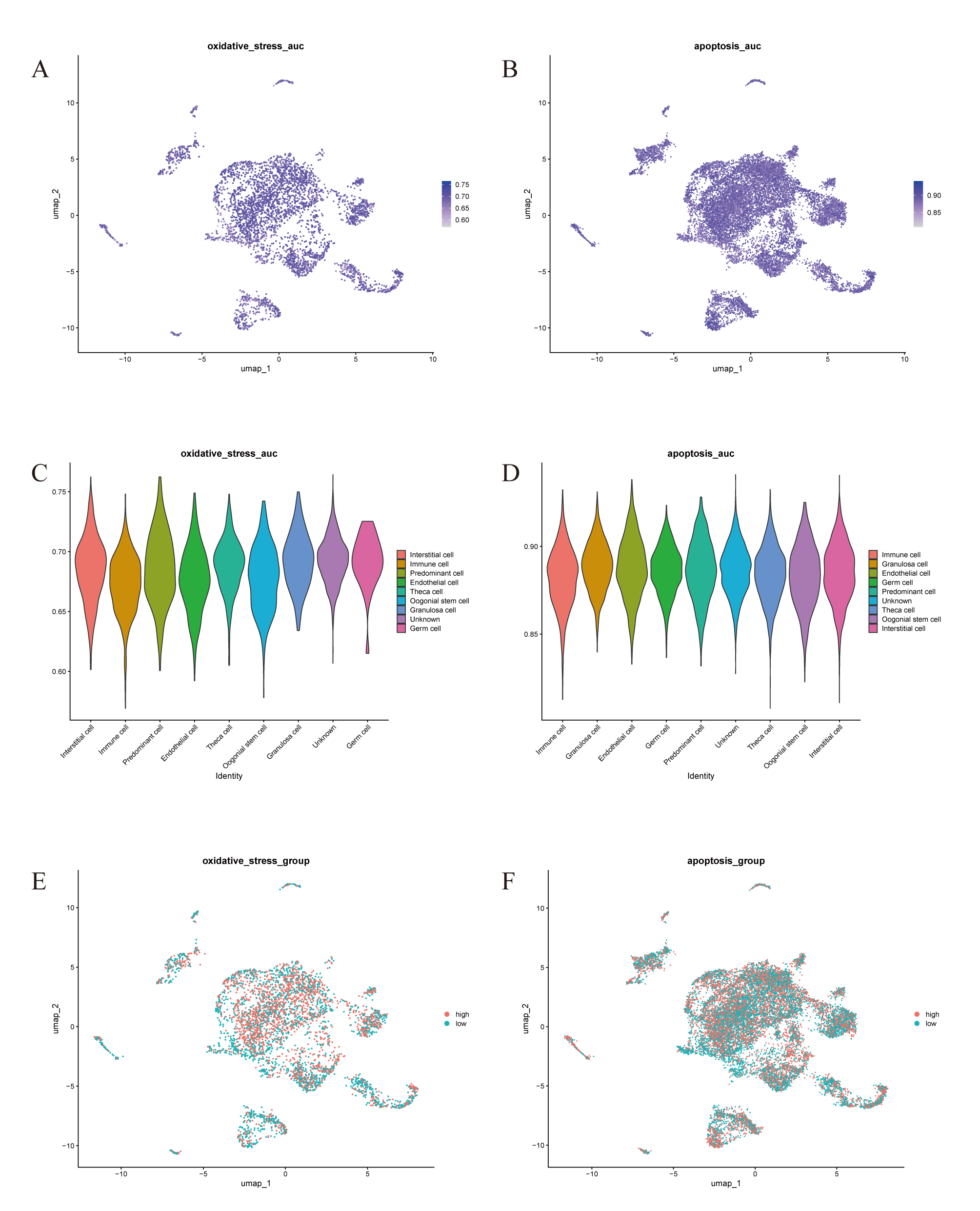

Supplement: Supplementary file 7 [file Image7.tif]

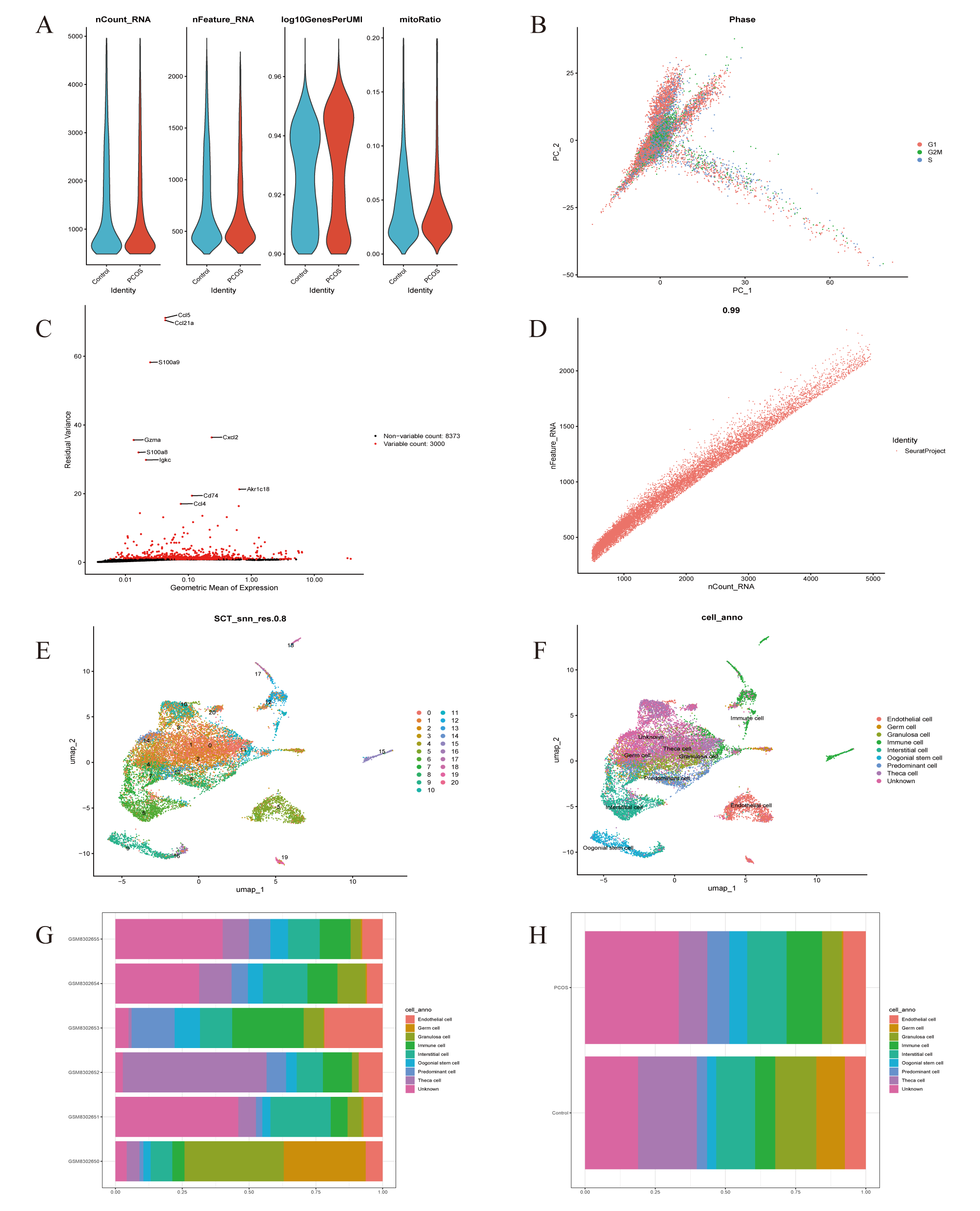

Supplement: Supplementary file 8 [file Image5.tif]
