# Supplementary material for: Network toxicology and single-cell analysis reveal key gene-mediated bisphenol a interference with granulosa cell function in polycystic ovary syndrome
Source: Front Pharmacol. 2026 Mar 2;17:1754568. doi: 10.3389/fphar.2026.1754568 (PMC12989541; doi:10.3389/fphar.2026.1754568)
Supplement: Supplementary file 2 [file Supplementaryfile1.docx]

**Supplementary material**

**Supplementary Table S1.** Overlapping hub genes between BPA and PCOS

**Supplementary Table S2.** Functional enrichment results for BPA–PCOS overlapping hub genes

**Supplementary Table S3.** Baseline demographic and clinical characteristics of the clinical validation cohort (PCOS vs. controls)

| **Supplementary Table S1.** Overlapping hub genes between BPA and PCOS | |
| --- | --- |
| Alias | Gene |
| *ABCB6* | *ATP Binding Cassette Subfamily B Member 6* |
| *ADAMTS7* | *ADAM Metallopeptidase With Thrombospondin Type 1 Motif 7* |
| *ADARB1* | *Adenosine Deaminase, RNA Specific B1* |
| *ADGRD2* | *Adhesion G Protein-Coupled Receptor D2* |
| *ADM* | *Adrenomedullin* |
| *AKR1C1* | *Aldo-Keto Reductase Family 1 Member C1* |
| *AKR1C2* | *Aldo-Keto Reductase Family 1 Member C2* |
| *AKR1C3* | *Aldo-Keto Reductase Family 1 Member C3* |
| *ANLN* | *Anillin Actin Binding Protein* |
| *ARHGAP23* | *Rho GTPase Activating Protein 23* |
| *ARHGAP30* | *Rho GTPase Activating Protein 30* |
| *ASPM* | *Asparagine Rich Spindle Microtubule Associated Protein* |
| *ATF1* | *Activating Transcription Factor 1* |
| *AZGP1* | *Alpha-2-Glycoprotein 1, Zinc-Binding* |
| *BAX* | *BCL2 Associated X, Apoptosis Regulator* |
| *BBOF1* | *B Box Only Factor 1* |
| *BCL11A* | *B Cell Leukemia/Lymphoma 11A* |
| *BCL2* | *B Cell Leukemia/Lymphoma 2* |
| *BLOC1S3* | *Biogenesis Of Lysosome-Related Organelles Complex 1 Subunit 3* |
| *BMP15* | *Bone Morphogenetic Protein 15* |
| *CAMK2D* | *Calcium/Calmodulin-Dependent Protein Kinase II Delta* |
| *CARMIL3* | *Ca2+ And Calmodulin Regulated Microfilament Associated Protein 3* |
| *CCNB1* | *Cyclin B1* |
| *CCNE2* | *Cyclin E2* |
| *CDC6* | *Cell Division Cycle 6* |
| *CDH15* | *Cadherin 15* |
| *CEP55* | *Centrosomal Protein 55* |
| *CIMAP1B* | *Ciliopathy And Meiosis Associated Protein 1B* |
| *CKS2* | *Cell Cycle Protein Kinase Subunit 2* |
| *CLDN4* | *Claudin 4* |
| *CMTR2* | *Cytoplasmic 3' mRNA Terminal Reducase 2* |
| *CNTROB* | *Centaurin Beta* |
| *CYP19A1* | *Cytochrome P450 Family 19 Subfamily A Member 1* |
| *DACT3* | *Dishevelled Associated Activator Of Beta Catenin 3* |
| *DCBLD2* | *Discoidin, Cysteine-Rich, Blood Coagulation Factor VIII-Like Domain 2* |
| *DDX54* | *DEAD-Box Helicase 54* |
| *DLG4* | *Discs, Large Homolog 4 (Drosophila)* |
| *DMD* | *Dystrophin* |
| *ELAVL3* | *ELAV Like RNA Binding Protein 3* |
| *EMSY* | *EMSY Homolog (BRCA1 Interactor)* |
| *ERRFI1* | *ERBB Receptor Feedback Inhibitor 1* |
| *FBXO44* | *F-Box Protein 44* |
| *FGF18* | *Fibroblast Growth Factor 18* |
| *FLT4* | *Fms Like Tyrosine Kinase 4* |
| *FOXP3* | *Forkhead Box P3* |
| *FSHR* | *Follicle-Stimulating Hormone Receptor* |
| *FUT7* | *Fucosyltransferase 7* |
| *GAB1* | *GRB2 Associated Binding Protein 1* |
| *GDF9* | *Growth Differentiation Factor 9* |
| *GNAS* | *GNAS Complex Locus* |
| *GP1BB* | *Glycoprotein 1b Beta Chain* |
| *HACD1* | *3-Hydroxyacyl-CoA Dehydrogenase 1* |
| *HELLS* | *Helios, Lymphoid-Specific Helix-Loop-Helix Protein* |
| *HMMR* | *Hyaluronan Mediated Motility Receptor* |
| *HOOK3* | *Hook Protein 3* |
| *HSD3B1* | *Hydroxysteroid Dehydrogenase 3 Beta 1* |
| *HSD3B2* | *Hydroxysteroid Dehydrogenase 3 Beta 2* |
| *IGHG1* | *Immunoglobulin Heavy Constant Gamma 1* |
| *IL34* | *Interleukin 34* |
| *INS* | *Insulin* |
| *ISYNA1* | *Isoform Specific Neuraminidase 1* |
| *JSRP1* | *Junctophilin Repeat Protein 1* |
| *KICS2* | *KICSTOR Subunit 2* |
| *LARP4* | *La Ribonucleoprotein 4* |
| *LEP* | *Leptin* |
| *LHB* | *Luteinizing Hormone Beta Subunit* |
| *LIF* | *Leukemia Inhibitory Factor* |
| *LIFR* | *Leukemia Inhibitory Factor Receptor* |
| *LMNA* | *Lamin A/C* |
| *LPCAT2* | *Lysophosphatidylcholine Acyltransferase 2* |
| *LRATD1* | *Lecithin Retinol Acyltransferase Domain Containing 1* |
| *LSM5* | *Lymphoid Specific Membrane Protein 5* |
| *LTK* | *Leukocyte Tyrosine Kinase* |
| *MAD2L1* | *Mitotic Arrest Deficient 2 Like 1* |
| *MALAT1* | *Metastasis Associated Lung Adenocarcinoma Transcript 1* |
| *MANEA* | *Mannosidase Endo-Alpha* |
| *MAPK8IP3* | *Mitogen-Activated Protein Kinase 8 Interacting Protein 3* |
| *MAPRE3* | *Microtubule Associated Protein, End Binding 3* |
| *MGAT5B* | *Mannosyl (Alpha-1,6-)-Glycoprotein Beta-1,6-N-Acetylglucosaminyltransferase 5B* |
| *MORN1* | *Membrane Occupation And Recognition Nexus 1* |
| *MTNR1B* | *Melatonin Receptor 1B* |
| *MYBL1* | *v-Myb Myeloblastosis Viral Oncogene Homolog 1* |
| *NMNAT3* | *Nicotinamide Mononucleotide Adenylyltransferase 3* |
| *NOP53* | *Nucleolar Protein 53* |
| *NPB* | *Neuropeptide B* |
| *NRG1* | *Neuregulin 1* |
| *PBK* | *PDZ Binding Kinase* |
| *PDE3B* | *Phosphodiesterase 3B, cGMP Inhibited* |
| *PDLIM4* | *PDZ And LIM Domain Containing 4* |
| *PLEKHG5* | *Pleckstrin Homology Domain Containing G5* |
| *PNPLA2* | *Patatin Like Phospholipase Domain Containing 2* |
| *PRKD2* | *Protein Kinase D2* |
| *PTAFR* | *Platelet Activating Factor Receptor* |
| *PTGER3* | *Prostaglandin E Receptor 3* |
| *PWWP2B* | *PWWP Domain Containing 2B* |
| *RAB2A* | *RAB2A, Member RAS Oncogene Family* |
| *RACGAP1* | *Rac GTPase Activating Protein 1* |
| *RASL10B* | *RAS Like Family 10 Member B* |
| *RBP4* | *Retinol Binding Protein 4* |
| *REXO1* | *3'-5' Exoribonuclease 1* |
| *RHPN1* | *Rhophilin 1, Actin Binding Protein* |
| *RP2* | *Retinitis Pigmentosa 2* |
| *RPL37A* | *Ribosomal Protein L37A* |
| *RRM2* | *Ribonucleotide Reductase M2* |
| *RUNX3* | *Runt Related Transcription Factor 3* |
| *S100A7A* | *S100 Calcium Binding Protein A7A* |
| *S100P* | *S100 Calcium Binding Protein P* |
| *SAP30L* | *Sin3 Associated Protein 30 Like* |
| *SCNN1A* | *Sodium Channel Non Voltage Gated 1 Alpha Subunit* |
| *SCT* | *Secretin* |
| *SFTPC* | *Surfactant Protein C* |
| *SOX15* | *SRY-Box Transcription Factor 15* |
| *SPATA21* | *Spermatogenesis Associated 21* |
| *SPTBN4* | *Spectrin Beta Chain, Non Erythrocytic 4* |
| *SRD5A1* | *Steroid 5 Alpha-Reductase 1, 3-Oxo-5 Alpha-Steroid 4-Dehydrogenase* |
| *SRSF10* | *Serine And Arginine Rich Splicing Factor 10* |
| *STAR* | *Steroidogenic Acute Regulatory Protein* |
| *TAB1* | *TGF Beta Activated Kinase 1 Binding Protein 1* |
| *TBX1* | *T-Box Transcription Factor 1* |
| *TCF15* | *Transcription Factor 15* |
| *TEAD2* | *TEAD Domain Transcription Factor 2* |
| *TFRC* | *Transferrin Receptor* |
| *THAP3* | *THAP Domain Containing 3* |
| *TMEFF2* | *Transmembrane Protein With EGF Like And Two Frizzled Like Domains 2* |
| *TMEM151A* | *Transmembrane Protein 151A* |
| *TMEM151B* | *Transmembrane Protein 151B* |
| *TMF1* | *Transmembrane F4 Domain Containing 1* |
| *TNPO2* | *Transportin 2* |
| *TNRC6B* | *Trinucleotide Repeat Containing 6B* |
| *TONSL* | *Tonsoku Like Meiosis Regulator* |
| *TOP2A* | *Topoisomerase 2 Alpha* |
| *TRPV6* | *Transient Receptor Potential Vanilloid 6* |
| *TTK* | *TTK Protein Kinase* |
| *TTLL9* | *Tubulin Tyrosine Ligase Like 9* |
| *UBE2H* | *Ubiquitin Conjugating Enzyme E2H* |
| *ZMYM5* | *Zinc Finger MYM-Type 5* |
| *ZNF205* | *Zinc Finger Protein 205* |
| *ZNF430* | *Zinc Finger Protein 430* |
| *ZSWIM9* | *Zinc Finger SWIM-Type 9* |

| **Supplementary Table S2.** Functional enrichment results for BPA–PCOS overlapping hub genes | | | | | | |
| --- | --- | --- | --- | --- | --- | --- |
| Index | Name |  | P-value | Adjusted p-value | Odds Ratio | Combined score |
| **GO: Biological Process** | | | | | | |
| 1 | C21-steroid hormone metabolic process | GO:0008207 | 1.33E-10 | 3.75E-07 | 33.38 | 49.98 |
| 2 | progesterone metabolic process | GO:0042448 | 7.20E-10 | 1.01E-06 | 57.02 | 53.33 |
| 3 | gonad development | GO:0008406 | 7.56E-08 | 5.12E-05 | 8.13 | 21.54 |
| 4 | development of primary sexual characteristics | GO:0045137 | 9.53E-08 | 5.12E-05 | 7.96 | 21.01 |
| 5 | sex differentiation | GO:0007548 | 9.89E-08 | 5.12E-05 | 7.17 | 19.91 |
| 6 | hormone metabolic process | GO:0042445 | 1.09E-07 | 5.12E-05 | 7.86 | 20.71 |
| 7 | reproductive structure development | GO:0048608 | 1.92E-07 | 7.71E-05 | 6.76 | 18.52 |
| 8 | reproductive system development | GO:0061458 | 2.23E-07 | 7.83E-05 | 6.67 | 18.22 |
| 9 | steroid hormone biosynthetic process | GO:0120178 | 5.42E-07 | 1.69E-04 | 20.70 | 27.39 |
| 10 | male gonad development | GO:0008584 | 9.68E-07 | 2.62E-04 | 21.70 | 28.39 |
| **GO: Molecular Function** | | | | | | |
| 1 | steroid dehydrogenase activity | GO:0016229 | 1.61E-07 | 5.58E-05 | 25.12 | 31.59 |
| 2 | steroid dehydrogenase activity, acting on the CH-OH group of donors, NAD or NADP as acceptor | GO:0033764 | 2.96E-06 | 5.12E-04 | 22.72 | 24.91 |
| 3 | bile acid binding | GO:0032052 | 4.11E-05 | 4.74E-03 | 42.99 | 23.80 |
| 4 | alditol:NADP+ 1-oxidoreductase activity | GO:0004032 | 7.46E-05 | 5.28E-03 | 35.82 | 21.31 |
| 5 | oxidoreductase activity, acting on the CH-CH group of donors, NAD or NADP as acceptor | GO:0016628 | 7.63E-05 | 5.28E-03 | 18.03 | 17.18 |
| 6 | estradiol 17-beta-dehydrogenase [NAD(P)] activity | GO:0004303 | 3.17E-04 | 1.83E-02 | 22.62 | 15.74 |
| 7 | alcohol dehydrogenase (NADP+) activity | GO:0008106 | 5.67E-04 | 2.80E-02 | 18.68 | 13.71 |
| 8 | oxidoreductase activity, acting on the CH-CH group of donors | GO:0016627 | 8.87E-04 | 3.84E-02 | 9.60 | 9.96 |
| 9 | growth factor activity | GO:0008083 | 1.09E-03 | 4.18E-02 | 5.39 | 7.20 |
| 10 | aldo-keto reductase (NADP) activity | GO:0004033 | 1.25E-03 | 4.33E-02 | 14.31 | 11.14 |
| **KEGG** | | | | | | |
| 1 | Ovarian steroidogenesis (Endocrine system) | hsa04913 | 1.03E-11 | 2.16E-09 | 26.40 | 51.88 |
| 2 | Steroid hormone biosynthesis (Lipid metabolism) | hsa00140 | 7.93E-07 | 8.28E-05 | 14.59 | 23.59 |
| 3 | Aldosterone synthesis and secretion (Endocrine system) | hsa04925 | 1.57E-05 | 1.09E-03 | 9.35 | 15.49 |
| 4 | Regulation of lipolysis in adipocytes (Endocrine system) | hsa04923 | 1.23E-04 | 6.41E-03 | 10.83 | 13.44 |
| 5 | p53 signaling pathway (Cell growth and apoptosis) | hsa04115 | 3.81E-04 | 1.59E-02 | 8.50 | 10.56 |
| 6 | Cushing syndrome (Endocrine and metabolic disease) | hsa04934 | 1.78E-03 | 6.08E-02 | 4.96 | 6.36 |
| 7 | Cortisol synthesis and secretion (Endocrine system) | hsa04927 | 2.03E-03 | 6.08E-02 | 7.75 | 7.95 |
| 8 | Neuroactive ligand-receptor interaction (Signaling molecules and interaction) | hsa04080 | 3.44E-03 | 8.98E-02 | 3.18 | 4.11 |
| 9 | EGFR tyrosine kinase inhibitor resistance (Drug resistance: antineoplastic) | hsa01521 | 4.33E-03 | 1.01E-01 | 6.29 | 6.27 |
| 10 | Oocyte meiosis (Cell growth and apoptosis) | hsa04114 | 5.89E-03 | 1.23E-01 | 4.56 | 4.88 |

Note: Adjusted p values were corrected using Bonferroni correction (as described in Methods).

| **Supplementary Table S3.** Baseline demographic and clinical characteristics of the clinical validation cohort (PCOS vs. controls) | | | | |
| --- | --- | --- | --- | --- |
| Index | Control | PCOS | t/χ^2^ | P-value |
| No. Of cases | 6 | 6 |  |  |
| Age (years) | 32.50 ± 4.72 | 30.17 ± 6.31 | 0.725 | 0.485 |
| BMI (kg/m2) | 21.08 ± 1.96 | 22.69 ± 3.20 | -1.058 | 0.315 |
| Infertility Type |  |  | 1.333 | 0.567 |
| Primary Infertility | 2 (33.33%) | 4(66.67%) |  |  |
| Secondary Infertility | 4(66.67%) | 2(33.33%) |  |  |
| Infertility Duration(years) | 3.33 ± 2.07 | 2.67 ± 1.75 | 0.603 | 0.560 |
| FSH(mIU/ml) | 5.11 ± 0.47 | 4.45 ± 0.76 | 1.815 | 0.100 |
| LH(mIU/ml) | 3.26 ± 1.39 | 4.78 ± 1.19 | -2.036 | 0.069 |
| PRL(ng/ml) | 14.42 ± 5.74 | 19.20 ± 6.03 | -1.408 | 0.190 |
| E2(pg/ml) | 31.83 ± 16.98 | 19.50 ± 8.73 | 1.582 | 0.145 |
| P(ng/ml) | 0.34 ± 0.26 | 0.27 ± 0.11 | 0.583 | 0.573 |
| T(ng/dl) | 0.32 ± 0.11 | 0.34 ± 0.06 | -0.361 | 0.725 |
| AMH(ng/ml) | 3.36 ± 1.62 | 7.54 ± 3.39 | -2.717 | 0.022 |
| Total dose of Gn used | 2220.83 ± 640.19 | 1733.33 ± 554.68 | 1.410 | 0.189 |
| Total day of Gn used | 11.17 ± 1.47 | 10.67 ± 1.37 | 0.610 | 0.556 |
| On hCG injection day |  |  |  |  |
| Endometrial thickness(mm) | 10.85 ± 3.29 | 11.83 ± 2.19 | -0.010 | 0.992 |
| E2（pg/ml） | 2869.83 ± 1165.32 | 3661.17 ± 1369.47 | -1.078 | 0.306 |

Note: Values are presented as mean ± SD or n (%). Normality was assessed using the Shapiro–Wilk test and homogeneity of variances was confirmed before group comparisons. Because E2, AMH, and endometrial thickness on the hCG injection day deviated from normality (p < 0.05), these variables were compared using the Mann–Whitney U test; all other continuous variables were compared using a two-tailed Student’s t-test. Categorical variables (Infertility Type) were compared using Fisher’s exact test. All tests were two-sided, and p < 0.05 was considered statistically significant.
